# Exploring the Potential of Digital Twins in Cancer Treatment: A Narrative Review of Reviews

**DOI:** 10.3390/jcm14103574

**Published:** 2025-05-20

**Authors:** Daniele Giansanti, Sandra Morelli

**Affiliations:** Centre TISP, ISS, 00161 Rome, Italy; sandra.morelli@iss.it

**Keywords:** cancer, oncology, digital twins, AI, artificial intelligence

## Abstract

**Background:** Digital twin (DT) technology, integrated with artificial intelligence (AI) and machine learning (ML), holds significant potential to transform oncology care. By creating dynamic virtual replicas of patients, DTs allow clinicians to simulate disease progression and treatment responses, offering a personalized approach to cancer treatment. **Aim:** This narrative review aimed to synthesize existing review studies on the application of digital twins in oncology, focusing on their potential benefits, challenges, and ethical considerations. **Methods:** The narrative review of reviews (NRR) followed a structured selection process using a standardized checklist. Searches were conducted in PubMed and Scopus with a predefined query on digital twins in oncology. Reviews were prioritized based on their synthesis of prior studies, with a focus on digital twins in oncology. Studies were evaluated using quality parameters (clear rationale, research design, methodology, results, conclusions, and conflict disclosure). Only studies with scores above a prefixed threshold and disclosed conflicts of interest were included in the final synthesis; seventeen studies were selected. **Results and Discussion:** DTs in oncology offer advantages such as enhanced decision-making, optimized treatment regimens, and improved clinical trial design. Moreover, economic forecasts suggest that the integration of digital twins into healthcare systems may significantly reduce treatment costs and drive growth in the precision medicine market. However, challenges include data integration issues, the complexity of biological modeling, and the need for robust computational resources. A comparison to cutting-edge research studies contributes to this direction and confirms also that ethical and legal considerations, particularly concerning AI, data privacy, and accountability, remain significant barriers. **Conclusions:** The integration of digital twins in oncology holds great promise, but requires careful attention to ethical, legal, and operational challenges. Multidisciplinary efforts, supported by evolving regulatory frameworks like those in the EU, are essential for ensuring responsible and effective implementation to improve patient outcomes.

## 1. Introduction

### 1.1. Digital Twins: Introductory Overview from Industry to Precision Oncology—Revolutionizing Healthcare Through Virtual Modeling

Digital twins (DTs) are interactive virtual replicas of physical entities, processes, or systems, continuously updated through real-time data from sensors, IoT devices, and other monitoring sources. They enable performance analysis, failure prediction, and system optimization through simulations, machine learning, and AI-driven insights. While digital twins originated in industrial and aerospace domains, their use has now expanded to sectors such as healthcare, manufacturing, transportation, and urban infrastructure. A variety of institutions and frameworks have offered definitions of digital twins [1,2,3,4], each emphasizing distinct features such as simulation, real-time data flow, or bidirectional interaction between the physical and virtual systems. Table 1 summarizes and compares prominent definitions.

These definitions collectively underscore the dynamic, real-time, and predictive nature of digital twins. From this foundation, DTs have emerged as a central technology in the digital transformation of multiple industries.

Across sectors, the adoption of DTs has catalyzed efficiency gains, cost reductions, and better-informed decision-making [5]). In manufacturing, DTs optimize production lines and enable predictive maintenance, reducing downtime and extending equipment lifespan. In transportation, they support real-time modeling of mobility systems to improve traffic flow and sustainability, as well as infrastructure resilience. In smart cities, digital twins help manage energy use, forecast demand, and test development scenarios virtually.

In healthcare, digital twins are gaining traction as a tool for personalized and adaptive medicine. The principles of continuous data integration and system simulation, already proven in industrial applications, are being repurposed to represent patient-specific biological processes. As with predictive maintenance in manufacturing, DTs in healthcare offer the potential to predict disease trajectories, simulate treatment responses, and guide clinical decisions dynamically.

Digital twins are closely tied to Industry 4.0, which emphasizes cyber–physical system integration, automation, and data-driven decision-making. These principles enable digital twins to function as living models, adapting continuously to real-world changes through bidirectional feedback loops between physical systems and their digital counterparts.

The growing relevance of digital twins in precision medicine is supported by evidence of clinical value [6,7,8]. For example, DTs have demonstrated efficacy in enhancing care for conditions like cancer, type 2 diabetes, and heart failure [6], enabling risk stratification, therapy effect simulation, and personalized health planning. Despite challenges such as data interoperability and privacy concerns [8], the field is advancing rapidly, supported by developments in AI and data science [7].

Specific implementations—such as digital twin systems (DTS) in pediatric chronic care [9] and virtual metabolic models for type 1 diabetes [10]—highlight the feasibility and diversity of DT application potential. These systems showcase the potential to go beyond disease treatment, supporting dynamic, individualized care pathways.

Among clinical fields, oncology stands out as a particularly promising area for DT implementation. Cancer care is inherently complex, requiring personalized and adaptive treatment strategies that respond dynamically to tumor heterogeneity and evolving patient conditions. Digital twins can help address these challenges by creating virtual representations of individual patients, continuously updated with clinical data, imaging, biomarkers, and treatment responses.

Given the rapid advancements in both digital health and precision oncology, DTs are increasingly positioned to improve patient outcomes by enabling individualized, data-driven care. Their integration allows for more precise, personalized interventions, enhancing treatment effectiveness and minimizing adverse effects. This potential is underscored by a growing body of literature [6,7,8,11,12,13,14,15,16,17,18,19], which highlights how DTs are poised to revolutionize cancer care.

Digital twins in oncology simulate treatment outcomes by integrating real-time data such as tumor characteristics, genomic profiles, and physiological parameters. These simulations allow clinicians to forecast how a patient’s disease may progress and how they might respond to various therapeutic strategies. For instance, Chaudhuri et al. (2023) [16] employed a predictive digital twin to optimize radiotherapy regimens for high-grade gliomas, enabling the fine-tuning of radiation doses to maximize tumor control while minimizing collateral damage to healthy tissue. Similarly, Hernandez-Boussard et al. (2021) [12] showed how digital twins can simulate cancer progression and treatment effects, supporting more nuanced and effective clinical decisions.

A personalized approach, in the context of digital twins, refers to tailoring treatment plans based on a patient’s unique tumor biology and overall health status. By modeling these parameters virtually, oncologists can evaluate the likely response to chemotherapy, immunotherapy, or radiation before initiating real-world treatment. This proactive modeling helps reduce side effects and increase therapeutic efficacy, advancing the aims of precision oncology, where interventions are designed with the individual patient at the center.

The impact of DTs becomes even more significant when aligned with the principles of precision oncology. As noted in [7], DTs integrate real-time monitoring, genetic insights, and clinical data to personalize therapies, minimizing risks while enhancing treatment outcomes. This level of precision has already shown promise in improving survival rates and quality of life.

Beyond clinical applications, digital twins are accelerating oncology research. As highlighted by [8], the convergence of artificial intelligence, machine learning, and big data analytics with DT frameworks has opened new frontiers—enabling drug discovery, predictive modeling, and surgical planning in silico. These tools support interdisciplinary collaboration and data-driven innovation, bringing novel therapies to patients faster and more efficiently.

DTs also support preventive and preemptive strategies. By simulating cancer risk based on genetic predisposition and environmental factors, digital twins allow for early detection and intervention. This proactive approach has the potential to slow disease progression, reduce costs, and increase survival rates. The ability to safely test therapeutic hypotheses in virtual environments fosters therapeutic innovation, accelerating timelines that would otherwise be limited by conventional clinical trials.

Moreover, digital twins are transforming clinical trial design. As described in [6], DTs simulate patient responses across virtual populations, streamlining trial selection, protocol optimization, and drug approval processes. These capabilities can significantly reduce time-to-market for new treatments, ultimately benefiting patients with earlier access to cutting-edge therapies.

DTs are especially valuable in scenarios of treatment uncertainty and complexity, such as in high-grade gliomas. Sager (2023) [11] demonstrated how DTs help fine-tune radiation regimens based on tumor response, improving precision and minimizing toxicity. Likewise, Hernandez-Boussard et al. (2021) [12] emphasized the value of predictive models for recurrence and metastasis, supporting risk stratification and patient-specific planning.

In personalized medicine, DTs are instrumental in refining individual therapeutic strategies. Shen et al. (2025) [14] illustrated how DTs can simulate tumor response across multiple modalities—including immunotherapy, chemotherapy, and radiation—enabling clinicians to develop bespoke treatment plans that improve outcomes and reduce adverse effects. These models update in real-time, enabling dynamic adaptation as a patient’s condition evolves.

Digital twins also support real-time monitoring. By continually assimilating new patient data, clinicians can adjust treatment protocols as needed. This is critical in oncology, where responsiveness to therapy often varies over time and between individuals.

Clinical trial methodologies are also evolving. According to a recent blog by the National Cancer Institute [13], DTs are now used to simulate trial outcomes in silico, optimizing study designs and accelerating the evaluation of clinical interventions. This shift reduces dependency on traditional, time-consuming methodologies.

Chaudhuri et al. (2023) [16] offered further validation by showing how predictive DTs can model radiotherapy regimens in glioma patients under uncertainty, improving the balance between efficacy and safety. Meanwhile, Shen et al. (2025) [14] demonstrated the use of high-fidelity virtual tumor models to simulate treatment outcomes, providing clinicians with granular insight into tumor dynamics. Likewise, [15] emphasized how radiation oncology is benefiting from multiscale modeling and encrypted data integration, reinforcing the reliability and scalability of DTs.

As these technologies mature, regulatory and ethical frameworks are becoming essential. Recent discussions [17,18] underscore the need to align DTs with clinical standards and regulatory protocols to ensure safety, accuracy, and ethical compliance. These frameworks will underpin the scaling and formal adoption of digital twins in healthcare.

The European Commission’s strategic vision [18] and recent guidance from health innovation bodies [19] point to digital twins as transformative tools in clinical trials and therapeutic design. Their inclusion in trial protocols could shorten development cycles and enhance the robustness of approvals, ultimately accelerating patient access to life-saving therapies.

Overall, the integration of digital twins in oncology is a transformative development with far-reaching implications for diagnosis, treatment, and research. By supporting precision medicine, enabling adaptive treatment, and accelerating innovation, DTs are reshaping the landscape of cancer care. Their continued evolution promises more personalized, effective, and data-driven therapies, offering new hope for patients and clinicians alike.

As illustrated in Table 2 below, digital twins have diverse applications across various domains in oncology, ranging from personalized treatment optimization in radiotherapy to enhancing early diagnosis through advanced imaging techniques, as highlighted in the analyzed references [6,7,8,9,10,11,12,13,14,15,16,17,18,19]. These applications are pivotal in driving the shift toward more data-driven, precise, and individualized cancer treatments.

### 1.2. Purpose

The integration of DT technology in oncology has gained significant attention in recent years, offering promising potential for personalized cancer care. Given the rapid pace of development and the increasing volume of publications on this subject, it is important to take a step back and analyze the overall landscape. This study aims to provide a narrative review of reviews (NRR) that synthesizes the current state of the integration of digital twins in oncology. This approach allows us to explore a wide range of insights and perspectives from the existing literature, providing a comprehensive understanding of the field without being constrained by the strict methodologies of a systematic review.

The general aim of this study is to highlight key themes, trends, and challenges in the integration of digital twins in cancer care, with a particular focus on how these technologies are being applied to improve treatment outcomes. Specifically, the objectives of this study are as follows:

- Analyze the overall bibliometric trends in the field: This will involve an exploration of the volume and development of research on digital twins in oncology over time, identifying key publications, authors, and influential sources that have shaped the field.
- Identify established themes and categories: The review will identify the main areas of focus across the literature, such as personalized treatment approaches, modeling of cancer progression, predictive analytics, and the role of artificial intelligence in enhancing cancer care.
- Examine opportunities and challenges: This section will explore the potential benefits of integrating digital twins in oncology, including more accurate treatment planning and improved patient outcomes, while also addressing challenges such as data privacy concerns, technological barriers, and the integration of digital twins into clinical practice.

By taking this narrative approach, the review aims to provide a broad and flexible examination of the evolution of digital twin technology in oncology. It will highlight the key developments, current applications, and future research directions in this transformative field, offering a holistic perspective on its potential to revolutionize cancer care.

## 2. Methods

The narrative review of review (NRR) followed a structured selection process based on a standardized narrative checklist (available online at the link [20]). Searches were conducted using PubMed and Scopus with predefined composite search keys. The qualification methodology employed used quality parameters described previously to determine the inclusion criteria for studies (Algorithm 1).
**Algorithm 1:** Selection Process for Narrative Review on Digital Twins in Oncology**Define search query:** (“(digital twins[Title/Abstract]) AND ((oncology[Title/Abstract]) OR (cancer[Title/Abstract]) OR (tumor[Title/Abstract])) “)2.**Conduct searches in PubMed and Scopus using the defined query.** 3.**Select relevant studies from peer-reviewed journals.** The reviews selected for this work center on the intersection of oncology and digital twin technology. Priority was given to the following:Recent review articles that synthesize findings from earlier studies, including previous reviews, offering an updated and integrated perspective on the evolving field.Comprehensive analyses that combine prior research insights with a specific emphasis on the application of DTs in oncology.To ensure alignment with the journal’s focus, articles primarily technical or centered on computer science—without clear clinical or translational implications—were excluded.4.**Evaluate each study based on the following parameters:****N1: Clear rationale in the introduction** The study should clearly explain the background, objectives, and the significance of the research question. The introduction must establish why the study is important and how it contributes to advancing knowledge in the field.**N2: Adequate research design** The design should be well suited to address the research question. This includes clear definitions of the study’s variables, appropriate control groups (if applicable), and a design that enables the study to produce valid, reliable results.**N3: Clearly described methodology** The methods used in the study must be explicitly outlined, with sufficient detail on how data were collected, analyzed, and interpreted. This allows other researchers to replicate or build upon the work.**N4: Well-presented results** The results should be clearly presented, with sufficient data to support conclusions. This includes appropriate use of tables, figures, and statistical analysis to highlight key findings.**N5: Conclusions justified by the results** The study’s conclusions should be logically derived from the results. Authors should refrain from making unsubstantiated claims, and their interpretation of the findings should be reasonable and supported by evidence.**N6: Disclosure of conflicts of interest** The study should explicitly disclose any potential conflicts of interest, including financial or personal interests that could have influenced the study’s design, conduct, or interpretation of results.5.**Assign scores to parameters N1-N5 on a scale from 1 to 5.** 
Each parameter is rated on a 5-point scale, where○**1** = Poor○**2** = Fair○**3** = Good○**4** = Very Good○**5** = ExcellentThis scale helps assess the overall quality of each study based on these key criteria.6.**Assess N6 using a binary Yes/No measure.** For conflicts of interest (N6), the evaluation is binary:○**Yes** = The study discloses any potential conflicts of interest.○**No** = The study does not disclose conflicts of interest.
7.**Preselect studies that meet the following criteria:** To ensure that only studies with a high level of methodological rigor and transparency are included, preselect studies based on the following criteria:○**N6 = Yes** (conflicts of interest disclosed).○**N1–N5 scores > 3** (ensuring sufficient methodological quality).
8.**Include preselected studies in the final synthesis.** Only studies that meet the above criteria (N6 = “Yes” and N1–N5 scores > 3) will be included in the final synthesis for review. This ensures the inclusion of studies with strong scientific integrity and relevance.

The initial search yielded a total of 71 reviews. From these, 33 studies were excluded due to their lack of direct focus. Following the evaluation according to the methodology described above, 17 review studies were retained [21,22,23,24,25,26,27,28,29,30,31,32,33,34,35,36,37] for further consideration, while the other ones were excluded.

## 3. Results

The study results are organized into two main sections to provide a structured and in-depth overview of the integration of digital twins (DTs) in oncology.

Section 3.1*:* This section provides an integrated overview of the current landscape of research on digital twins (DTs) in oncology. It first analyzes bibliometric trends by examining the progression of publication volumes over time. This analysis highlights the growing research interest in the application of DT technology in cancer care, identifying key milestones and shifts in scientific focus. Furthermore, the section examines selected review studies to explore emerging research themes, such as personalized cancer treatment, predictive modeling of disease progression, and the use of DTs to optimize therapy responses. Together, these insights offer a comprehensive understanding of the evolution, innovations, and key directions shaping the field of digital twins in oncology.

Section 3.2*:* Based on the findings from the analyzed studies, this section identifies emerging opportunities for the application of digital twins in oncology, such as more precise treatment planning and improved patient outcomes. It also highlights the barriers to wider implementation, including challenges related to data interoperability, technological adoption, and ethical concerns, and emphasizes areas requiring further research and development to drive significant advancements in the field.

This systematic approach provides a clear and comprehensive view of the current state of digital twin technology in oncology, shedding light on ongoing trends and future prospects for transforming cancer care.

### 3.1. Research Trends and Thematic Insights on Digital Twins in Oncology

Section 3.1 is organized into two subsections that collectively examine the trends and emerging themes in the application of digital twins (DTs) in oncology:

Section 3.1.1 Trends: This subsection delves into the bibliometric trends by analyzing the volume and growth of publications over time. It begins with a PubMed search that identifies 73 studies since 2019, focusing on digital twins in oncology. The analysis highlights that the majority of these studies (97.26%) have been published within the last five years. Additionally, it explores the proportion of review articles (23.29%) and compares oncology-related publications with those in the broader health domain. This section also presents visual data to emphasize the rapid increase in research on digital twins in oncology, underscoring the growing interest in the field. The trends indicate a significant shift in focus towards oncology, with a growing number of studies synthesizing existing knowledge.

Section 3.1.2 Emerging Themes and Categorization: This subsection examines the selected review studies on digital twins in oncology, categorizing the emerging themes and identifying key areas of research. The integration of artificial intelligence (AI) and machine learning (ML) with digital twins is highlighted as a crucial development for personalized cancer treatment. The section discusses how DTs enable personalized care by simulating disease progression, treatment responses, and therapeutic outcomes. It reviews various studies that have shown advancements in modeling tumor dynamics, optimizing therapy responses, and improving clinical decision-making. A synthesis table (Table 1) is provided to give an overview of the studies, categorizing them by focus area (e.g., personalized treatment, predictive modeling) and innovative approaches. This subsection further emphasizes the transformative potential of digital twins in oncology, demonstrating how these technologies are revolutionizing cancer care through tailored treatments and enhanced clinical predictions.

In this section, we present both the quantitative growth of publications on digital twins in oncology and the qualitative advancements in the research. This combined approach provides a holistic view of the development of digital twin technologies in oncology, shedding light on both the trends in publication and the innovation occurring in the field.

#### 3.1.1. Trends

**Figure 1:**
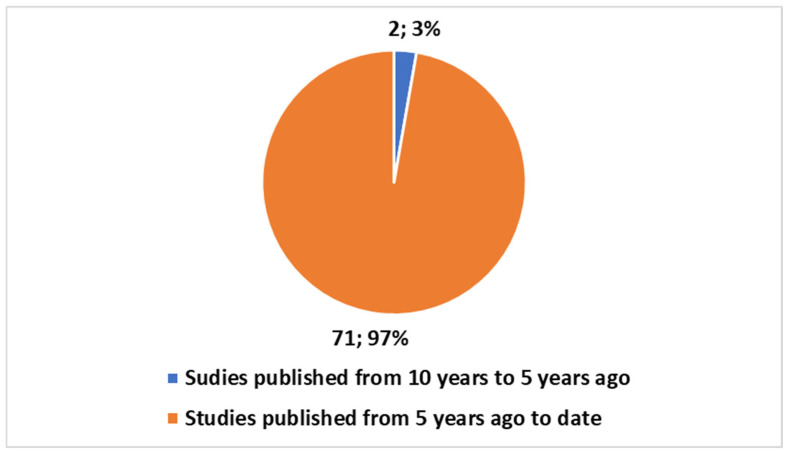
The number of publications on digital twins (DTs) in oncology in the last five years and between the last ten and five years. The graph illustrates the sharp increase in publications on DTs in oncology over the past five years, emphasizing the growing interest in the application of DT technology in cancer research.

**Figure 2:**
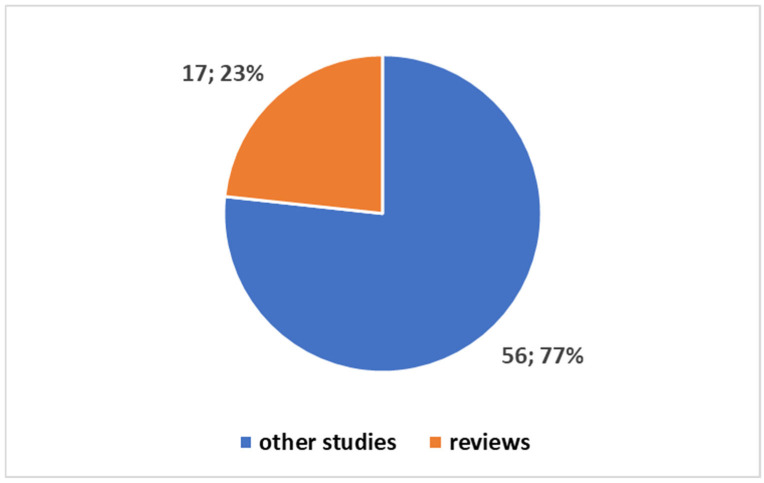
Distribution between review articles and other primary studies on DTs in oncology. This figure highlights the proportion of review articles (23.29%) within the oncology-related publications on digital twins, providing insights into the synthesis and consolidation of research in this field.

A PubMed search using the search term from Box 1 yielded 73 studies since 2019, focusing on the application of digital twins in oncology. When narrowing the timeframe to the last five years, it becomes evident that 71 of these studies, representing 97.26% of the total, have been published in the last five years (Figure 1). Of these 73 studies, 17 (or 23.29%) are review articles, with no systematic reviews present. All 17 reviews have been published within the last five years (Figure 2).

Box 1 **Used search keys.**

*(digital twins[Title/Abstract]) AND ((oncology[Title/Abstract]) OR (cancer[Title/Abstract]) OR (tumor[Title/Abstract]))* *(digital twins[Title/Abstract])* 

Figure 1 illustrates the number of publications on DTs in oncology within the last five years and between the last ten and five years. The data for this chart was extracted and plotted using Excel, with two categories: publications from the last five years and those from the period between five to ten years ago. The publication numbers for each category were visually represented using a pie chart, with percentages calculated based on the total count of 73 studies.

Figure 2 shows the distribution between review articles and other primary studies in the field of DTs in oncology. This pie chart was created in Excel, where the proportion of review articles (17 studies) compared to primary research (56 studies) was calculated and depicted visually. The chart reflects the fact that all review articles were published in the last five years, representing a significant focus on synthesizing existing research in the field.

**Figure 3:**
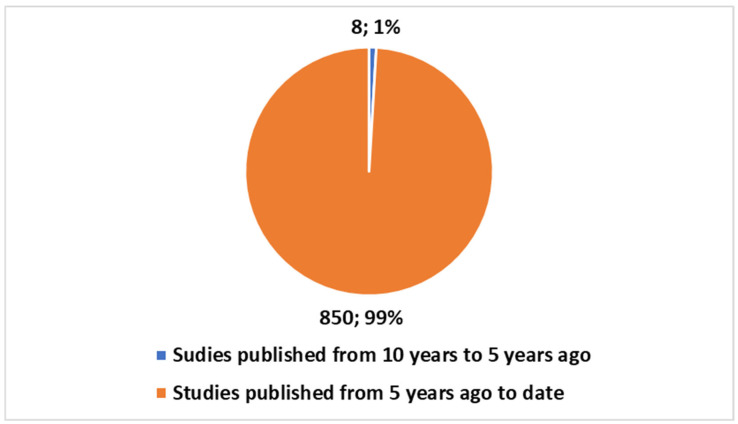
The number of publications on digital twins (DTs) in the health domain in the last five years and between the last ten and five years. The graph shows a significant rise in DT publications across all health domains in the past five years, indicating an overall surge in the integration of DTs in medical research.

**Figure 4:**
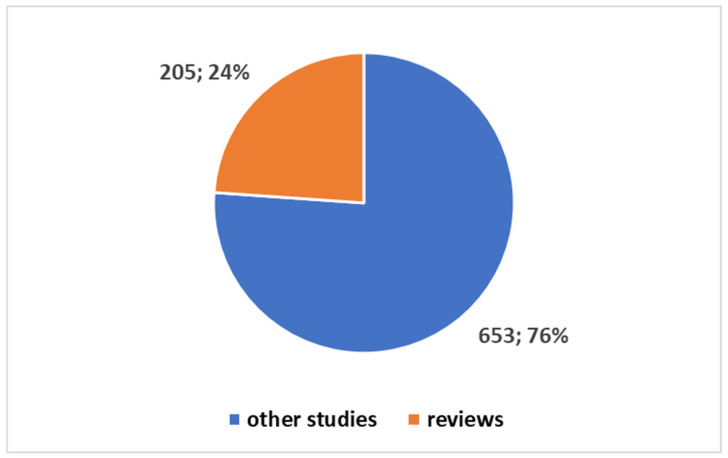
Distribution between review articles and other primary studies in digital twin research across the health domain. This figure compares the percentage of review articles (23.91%) in the broader health domain, showing a similar focus on synthesizing research findings across different health-related applications of DTs.

When comparing the total publications on DTs in the health domain using the second search term from Box 1, we find 858 publications since 2016, which means all have been produced in the last 10 years. Out of these, 850 publications (representing 98.6%) were published in the last five years (Figure 3). Additionally, a total of 205 (or 23.91%) are reviews (Figure 4). Both figures were generated in Excel using the same methodology as in Figure 1 and Figure 2. The bar chart in Figure 3 shows the same two time categories (last five years and five to ten years ago) for the entire health domain dataset, with numbers and percentages visually represented. Figure 4, similarly, displays the distribution of review articles (205) and primary studies (653) within the broader health domain using a pie chart format.

Figure 3 depicts the number of publications on DTs in the last five years and between the last ten and five years, visually comparing oncology-related publications (73) with those in the broader health domain (858). The pie chart was built in Excel, following the same structure as Figure 1, ensuring a consistent comparison of trends over the specified timeframes.

Figure 4 illustrates the distribution between review articles and other primary studies in the health domain. This pie chart was also created in Excel and highlights the proportion of reviews within the health domain (23.91%) compared to primary studies (76.09%).

When we compare the 73 oncology-related studies with the 858 publications focused on DTs in the health domain, we observe that the oncology-related publications account for approximately 8.5% of the total. The percentage of review articles in oncology (23.29%) is comparable to the broader health domain (23.91%), highlighting a similar level of focus on synthesizing existing research in both areas. While oncology accounts for 8.5% of the total publications on DTs in health, the relatively high proportion of review articles in both oncology and the overall health domain suggests a growing interest in evaluating and consolidating research findings. This could point to a maturation of both fields, where researchers aim to summarize and refine the knowledge base to better understand the impact and potential of DTs.

#### 3.1.2. Emerging Themes and Categorization

##### Emerging Themes

The selected review studies on the application of digital twins in oncology [21,22,23,24,25,26,27,28,29,30,31,32,33,34,35,36,37] were analyzed. Generally, the NRR highlights that the digital twin (DT) technology, when integrated with artificial intelligence (AI) and machine learning (ML), has opened new frontiers in personalized oncology care. This integration holds the potential to significantly improve both the precision of cancer treatments and the clinical decision-making process. The ability to create dynamic virtual replicas of patients and simulate disease progression, treatment responses, and therapeutic outcomes provides a tailored approach that is increasingly seen as the future of cancer treatment.

Table 3, “Synthesis of Digital Twin Applications and Innovative Approaches in Oncology”, is designed to provide a systematic overview of the various ways digital twin (DT) technology is being applied to oncology. It summarizes key studies that explore the integration of digital twin concepts into different aspects of cancer care, emphasizing innovative approaches and the enhancement of clinical decision-making, personalized treatments, and optimized therapeutic outcomes.

The table is organized into four main columns, each serving a specific purpose:

1. **Reference**: This column reports the references.
2. **Brief Description**: This section provides a concise summary of the study’s key focus or contribution. It outlines the primary research questions or objectives of the study and the general approach used to explore the integration of digital twins in oncology.
3. **Focus on Oncology**: Here, the table specifies how each study is particularly relevant to oncology. It highlights the specific oncological areas or challenges addressed in the study, such as cancer treatment personalization, tumor progression prediction, or optimizing radiotherapy. This column helps to contextualize the research within the specific needs of oncology.
4. **Innovative Approach**: This final column summarizes the novel contributions or methodologies introduced in each study. It emphasizes how digital twin technology or related innovations, such as AI and machine learning, are being utilized to enhance clinical decision-making, personalize treatment plans, or improve cancer care outcomes.

By synthesizing these studies, the table highlights the diverse applications of digital twin technology in oncology and underscores the innovative potential of this emerging field in shaping personalized cancer care strategies.

As reported in the table, the research outlined in [21] demonstrates how machine learning-based models are crucial for the validation and clinical utility of DTs in oncology. These models can process vast amounts of clinical data, supporting decisions that enhance patient care. Similarly, [22,23] delve deeper into how DTs, particularly when paired with real-time simulations, enable personalized cancer treatments, addressing challenges such as data integration and patient variability. A notable breakthrough in DT research, discussed in [24], focuses on the role of diagnostic simulations and predictive modeling in oncology. This approach enables clinicians to visualize patient-specific scenarios, ensuring that treatment plans are precisely tailored to individual needs. Ref. [25] emphasized the necessity of ensuring the reliability and safety of these digital models through verification, validation, and uncertainty quantification (VVUQ), making them clinically applicable. Oncology-specific applications are expanding, as explored in [26,27], where DTs are used for personalized diagnostics, therapy simulations, and cancer progression modeling. These advancements are further supported by [28], which discusses the potential of generative digital twins (GDTs) in creating high-fidelity tumor replicas, advancing precision diagnostics and personalized treatment pathways. Radiotherapy has seen notable improvements through the integration of DTs. Ref. [29] illustrated how patient-specific digital twins can guide personalized radiotherapy, optimizing tumor control while minimizing damage to healthy tissues. Additionally, in immuno-oncology, Ref. [30] focused on the creation of DTs for more personalized cancer treatments, offering solutions to challenges in virtual patient population generation and data integration. Theranostic digital twins (TDTs), highlighted in [31], have demonstrated their capacity to personalize radiopharmaceutical therapy, optimizing radiation doses and minimizing toxicity, thus improving treatment safety. The use of mechanistic learning, as described in [32], further enhances the prediction of tumor responses, enabling more precise and effective treatments. The impact of DTs on clinical trials and personalized treatment strategies is well documented in [33,34]. These studies showcase the potential for DTs to simulate patient-specific interventions, improving clinical trial designs and therapeutic predictions. Furthermore, Ref. [34] discussed the application of optimal control theory and data assimilation techniques to refine clinical interventions and improve patient outcomes. The integration of DTs and AI/ML in drug development, as outlined in [35], is pivotal in oncology. The ability to incorporate multi-dimensional biomarker data into personalized treatment plans is helping create more accurate and effective cancer therapies. Ref. [36] extended this idea by emphasizing the potential of image-guided, mechanism-based DTs to simulate tumor dynamics, which is crucial for predicting tumor behavior and optimizing treatment plans. Furthermore, Ref. [37] highlighted how digital twins can be used to model complex diseases like inflammatory bowel disease (IBD), with significant implications for oncology. It shows how multiscale hybrid models, combining mechanistic approaches and machine learning, can predict therapeutic responses in IBD—a concept directly translatable to cancer care. By modeling specific biological pathways, such as IL-6 signaling, which is implicated in both IBD and colon cancer, the study underscores the potential of digital twins to improve precision medicine in oncology by enhancing the accuracy of treatment predictions. Overall, the growing body of research on digital twin technologies in oncology demonstrates their transformative potential in personalized medicine. By integrating AI, ML, and advanced computational techniques, DTs provide new avenues for tailoring cancer treatments to individual patients, improving clinical outcomes, and ultimately revolutionizing the way cancer care is delivered. Through continued innovation, digital twin technologies are poised to redefine the future of oncology. In oncology, the use of digital twins (DTs) is revolutionizing personalized treatment strategies. For treatment planning, DTs simulate disease progression and treatment pathways specific to each patient, allowing for tailored therapies that address individual needs [22]. This innovation is particularly impactful in areas like radiotherapy, where personalized digital twins guide treatment adaptations based on patient-specific data, ensuring optimal tumor control while minimizing harm to healthy tissue [29]. Moreover, theranostic digital twins (TDTs) are being employed to personalize radiopharmaceutical therapy by adjusting radiation doses to individual patient profiles, thus improving precision and minimizing side effects [31]. Additionally, the integration of AI and DTs in oncology is enhancing diagnostic accuracy and therapeutic effectiveness. For treatment planning, this combination is particularly evident in the use of imaging, pathology, radiotherapy, and genomics, where DTs help refine precision medicine, enabling better-targeted interventions [23]. Digital twins also play a pivotal role in optimizing clinical trial simulations by integrating patient-specific data and mathematical models. These tools help researchers refine treatment strategies, accelerate drug development, and ultimately improve patient outcomes [34]. Digital twins (DTs) enable the simulation of patient-specific clinical scenarios and the prediction of cancer progression, supporting personalized treatment planning. This capability extends to oncological applications that intersect with sub-domains of neuroscience, where integrating neurological insights enhances precision and contextual relevance [24]. In drug development, the use of digital twins has become instrumental in optimizing precision medicine by integrating real-world and -omics data, allowing for model-informed approaches that personalize treatments and improve drug development strategies [35]. For treatment planning, digital twins are increasingly being used to refine decision-making processes, ensuring treatments are tailored to individual patient profiles, which leads to better-targeted therapies. In drug development, digital twins offer unique advantages by simulating clinical trials and patient-specific responses to various treatment protocols. These simulations help identify the most effective therapies before they are tested in real-world settings, reducing the risk of failure and speeding up the development process. By utilizing patient-specific data in real-time, drug development can be tailored to meet the needs of diverse populations, thus improving therapeutic efficacy and minimizing adverse reactions. These advancements are contributing to more efficient, effective, and personalized cancer care, ensuring that therapies are better suited to each patient’s unique needs.

##### Categorization

DTs are rapidly emerging as a transformative technology in precision oncology, enabling more personalized treatments and optimized clinical outcomes. These virtual models simulate patient-specific biological systems and responses, offering novel opportunities for improving cancer care. This review categorizes recent advancements in DT research in oncology, highlighting several key areas as reported in Table 4.

Table 4, “Study Categorization”, is designed to provide a structured overview of the key themes and areas of research related to the application of digital twin (DT) technology in oncology. It organizes studies into distinct categories, each highlighting a specific aspect of digital twin research and its integration into the field of oncology. The table is composed of three main columns, each serving a distinct purpose:

1. **Category:** 
  This column organizes the studies into broader research areas, offering a high-level view of the different aspects of digital twin applications in oncology. Categories such as “Foundations and Frameworks,” “Innovative Applications,” “Challenges in Development,” and others help to contextualize the research, allowing readers to easily understand the focus of each study.

2. **Key Topics:** 
  The Key Topics column provides a detailed summary of the core subjects covered. It highlights the main concepts, methodologies, and areas of interest discussed in the research, such as computational frameworks, agent-based modeling, and real-time data assimilation. This section enables readers to quickly grasp the specific contributions of each study to the field of oncology.

3. **Key References:** 
  The Key References column lists the key studies and sources that underpin the research within each category.

The combination of these fields offers an organized and comprehensive way to explore the diverse and evolving applications of digital twin technology in oncology.

As illustrated in the table, the foundations and frameworks for DTs are essential, with computational models and mathematical frameworks explored to enhance personalized clinical trials and precision oncology. Notable contributions include the work of [27,34], which discussed computational frameworks for precision oncology using digital twins and data assimilation for personalized clinical trials. Additionally, Ref. [33] provided an overview of digital twin development in oncology, addressing challenges such as data integration and future advancements in personalized medicine.

In the realm of innovative applications, DTs are already being utilized for personalized treatment planning in radiotherapy [29] and theranostic treatments in radiopharmaceutical therapies [31]. Ref. [36] explored how medical imaging and tissue-scale modeling are being applied to create patient-specific digital twins, focusing on tumor dynamics and treatment optimization.

The integration of data, machine learning, and modeling is a crucial area of research, as AI and machine learning approaches enhance digital twin applications in oncology. Ref. [35] discussed model-informed precision medicine using AI/ML for drug development and treatment strategies, while [37] explored hybrid models combining AI and mechanistic modeling to improve therapy precision in both oncology and inflammatory bowel disease. Ref. [21] reviewed the integration of externally validated machine learning models in oncology to support clinical decision-making.

However, the development of DTs faces several challenges, particularly regarding data integration and biological complexity. Ref. [28] identified issues in generating spatially resolved generative digital twins, and [30] explored the difficulties in customizing immuno-oncology-specific models for patient treatment. Additionally, Ref. [26] addressed the complexity of cancer heterogeneity and integrating multi-modal data for accurate digital twin modeling.

The clinical utility and impact of DTs in oncology are increasingly recognized, with the potential to revolutionize clinical decision-making and treatment outcomes. Ref. [32] emphasized the role of mechanistic learning in improving treatment outcomes, while [24] discussed the potential of digital health twins to transform oncology care by leveraging big data, AI, and digital twin technology.

Looking ahead, future directions in DT development point to overcoming existing challenges such as privacy concerns and data integration. Refs. [27,33] highlighted the long-term potential of DTs in enhancing oncology treatment by using patient-specific data and computational modeling, with an outlook on transformative advancements.

Cross-disciplinary approaches are also critical to fully realize the potential of DTs in oncology. Ref. [37] examined the integration of hybrid AI models across multiple fields, including oncology and IBD, to enhance therapeutic responses, while [35] discussed how AI/ML and digital twins are reshaping clinical trial designs and patient-specific treatments.

Finally, personalized trials and patient-centered models are central to advancing cancer treatments. Ref. [34] outlined the importance of digital twins in personalized clinical trials, utilizing real-time data assimilation and mathematical models to optimize treatment plans. This synthesis offers a comprehensive overview of how digital twins are shaping the future of oncology, emphasizing both current advancements and future opportunities for personalized cancer care.

### 3.2. Opportunities and Challenges

The integration of digital twins (DTs) in oncology presents both exciting opportunities and significant challenges. Table 5, ”Opportunities and Challenges in the Oncology and Digital Twins Integration”, is designed to present a structured comparison of the potential benefits and obstacles in the use of digital twin (DT) technology in oncology. It is organized into two main columns: Opportunities and Barriers, which highlight the advantages and limitations of integrating DTs into oncology practice.

1. **Study:** 
  This column reports the references.

2. **Opportunities:** 
  The Opportunities column outlines the potential benefits of applying digital twin technology in oncology. It emphasizes how DTs can enhance clinical decision-making by providing personalized treatment options, predicting cancer treatment responses, and optimizing therapeutic strategies. This section highlights the innovations that digital twin technology brings to oncology, showcasing its value in improving patient care and treatment outcomes.

3. **Barriers:** 
  In contrast, the Barriers column identifies the challenges associated with the integration of DTs into oncology. These challenges include issues such as the variability of clinical data, the complexity of real-world clinical environments, and difficulties in validating DT models across diverse patient populations. It also addresses the technological and operational hurdles that need to be overcome to ensure the effective use of digital twin technology in cancer treatment.

Together, these columns provide a balanced view of both the advantages and the obstacles faced in the implementation of digital twin technology in oncology, allowing for a comprehensive understanding of the current state and the future potential of this integration. As illustrated in the table, DTs have the potential to revolutionize cancer treatment by enabling more personalized, accurate, and dynamic approaches to patient care. From improving decision-making processes and optimizing treatment strategies to personalizing radiotherapy and chemotherapy, DTs can facilitate a deeper understanding of tumor dynamics, individual patient responses, and therapeutic outcomes. However, as Table 3 demonstrates, there are numerous barriers to the wide-spread adoption of DTs in clinical oncology. Challenges such as data integration issues, the complexity of biological modeling, computational resource requirements, and clinical validation hurdles all need to be addressed for DTs to achieve their full potential. For example, in [21], the authors highlighted the difficulty in integrating heterogeneous clinical data to improve decision-making accuracy, while in [29], the ability to personalize radio-therapy treatment plans via DTs could be hindered by the reliance on high-quality imag-ing techniques that may not be universally available. A key opportunity emphasized in [27] is the use of agent-based models, where computational frameworks based on DTs can simulate patient-specific treatment responses to optimize therapy selection. This approach allows clinicians to predict how individual patients will react to various treatment options, offering a more precise, personalized care plan. Similarly, Ref. [35] underscored how integrating AI/ML with DTs can enhance precision in drug development, offering more targeted treatments and enabling better clinical trial designs. However, as noted in [30], the lack of detailed immune data may limit the application of DTs in immuno-oncology therapies, showcasing the complexity of building accurate and reliable models for cancer treatment. The use of DTs in personalized clinical trials, discussed in [34], provides an example of how these digital models can help tailor trials based on real-time data, improving patient recruitment and therapeutic outcomes. Yet, the need for real-time data integration, as mentioned in [34], presents computational challenges that may limit the scalability of DTs in large, diverse patient populations. Despite these challenges, the transformative potential of DTs in oncology remains significant. Moving forward, addressing the barriers associated with data quality, computational power, and clinical validation will be crucial in enabling DTs to reshape personalized cancer care and improve treatment outcomes on a broader scale.

## 4. Discussion

The discussion is organized into seven sections to comprehensively address the findings from the NRR and complement the gaps identified in the results.

Section 4.1: Highlights, Detected Gaps, and Added Value outlines the main findings and key gaps in the current research, laying the groundwork for further discussion. It identifies opportunities and challenges, highlighting the need for further exploration.

The following Section 4.2, Section 4.3, Section 4.4, Section 4.5 and Section 4.6 build logically upon these findings, with Section 4.2: The Evolution of Digital Twins: Market Expansion and Oncological Impact examining market expansion and oncology impact, which follows naturally from the gaps identified in Section 4.1. This sets the stage for exploring how digital twins (DTs) are expected to grow in the coming years, particularly in terms of their increasing integration within healthcare systems. As technology advances, the potential applications of DTs in oncology are expected to expand, offering new opportunities for precision medicine and personalized treatment strategies.

Subsequent sections focus on the contributions of cutting-edge research, ethical/legal complexities, available platforms, and future directions. Each of these follows from the needs and opportunities highlighted in Section 4.1, ensuring a coherent flow that connects the gaps with the potential solutions and emerging trends in the field.

Section 4.3: Comparison and Contribution of the Cutting-Edge Research compares the latest advances in digital twin research in oncology, highlighting contributions that push the boundaries of what DTs can achieve in oncology, with a focus on innovative methodologies and their clinical implications.

Section 4.4: Integrating Digital Twins: Navigating Ethical and Legal Complexities addresses the ethical and legal challenges associated with deploying DTs in oncology, including patient privacy, data ownership, and regulatory hurdles, and how these concerns can be mitigated.

Section 4.5: Core vs. Supportive Technologies in Digital Twins: Available Platforms and Their Roles in Oncology reports examples of devices/products available and discusses the distinction between core technologies (such as AI and modeling tools) and supportive platforms that complement digital twin development, examining their current roles in oncological settings.

Section 4.6: Future Directions briefly outlines the emerging trends and potential future advancements in digital twin technology, providing a forward-looking perspective on where the field is headed and the innovations needed to overcome current limitations.

The limitations of the study are discussed in Section 4.7.

*SYNOPTIC DIAGRAM*

**Figure 5:**
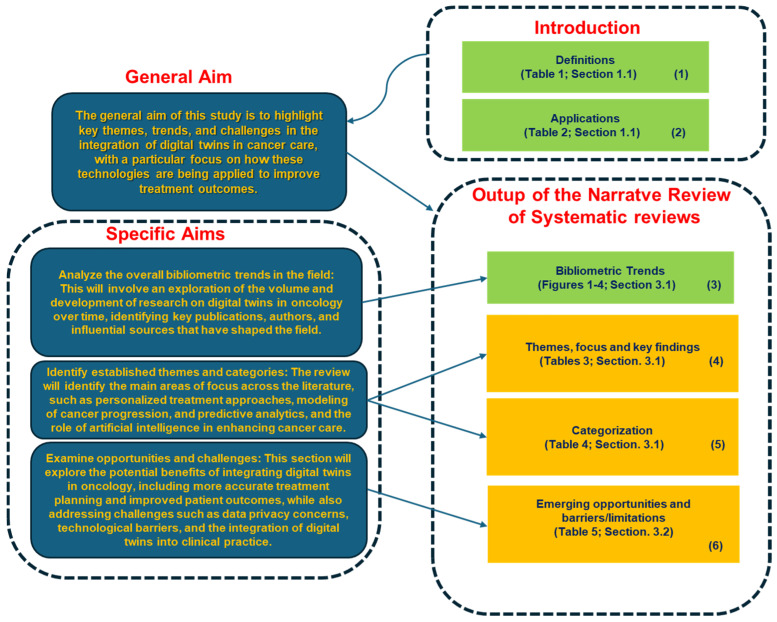
First synoptic diagram. (The number of blocks are reported for the sake of clarity during the explanation in the text).

**Figure 6:**
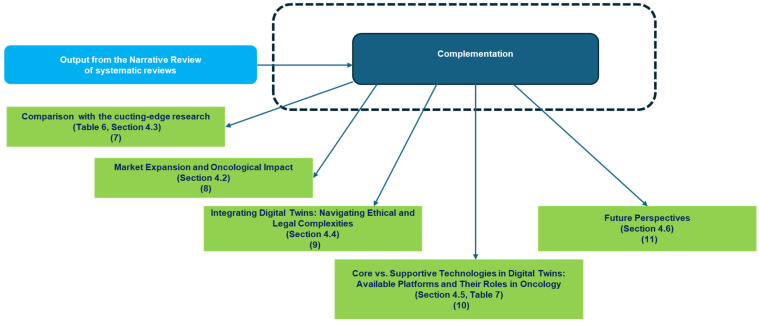
Second synoptic diagram. (The number of blocks are reported for the sake of clarity during the explanation in the text).

Figure 5 and Figure 6 present two synoptic diagrams that outline the rationale behind the design of the narrative review of reviews. These diagrams provide a structured visual representation of how the study was developed, showing the logical sequence of its different phases and how they interconnect.

**First Diagram (Figure 5** **): Linking Objectives to Analysis**

The first diagram (Figure 4) illustrates how the study was structured based on its general objective and three specific objectives. The logical progression follows a top-down approach:

- **Block 1 and 2**: These blocks recall the definitions (Table 1) and applications in oncology (Table 2) reported in the introductive discourse to anticipate the rationale for the NRR.
- **Block 3**: This block represents the bibliometric trends reported in Figure 1, Figure 2, Figure 3 and Figure 4 (Section 3.1). These trends were analyzed to provide an overview of the scientific production in the field.
- **Block 4**: This block corresponds to the identification of thematic areas, as presented in Table 3 (Section 3.1).
- **Block 5**: Building upon the thematic categorization, this block recalls the categorization as reported in Table 4 (Section 3.1), enabling a deeper understanding of the different ways DTs are applied in oncology.
- **Block 6**: This block synthesizes the opportunities and challenges identified in the reviewed studies, as reported in Table 5 (Section 3.2). These findings highlight both the potential benefits of SARSs’ applications in the health domain—and the barriers/limitations

This diagram provides a step-by-step visualization of the study’s methodological process, from bibliometric analysis to thematic categorization, comparative analysis, and the identification of emerging opportunities and challenges.

**Second Diagram (Figure 6** **): Connecting Discussion to Findings**

The second diagram (Figure 6) is logically connected to the first and illustrates how the study transitions from the findings of the literature review to the discussion based on the needed complementation discourse. The sequential organization follows a structured approach:

- **Block 7** reports a comparison with the cutting-edge research as discussed in Table 6 (Section 4.3).
- **Blocks 8 and 9** map the emerging market growth discussed in Section 4.2 and the ethical and legal deepening discussed in Section 4.4
- **Block 10** faces the devices and platforms detailing and discussing the Core vs. Supportive Technologies in Table 7 (Section 4.5).
- **Block 11** finally identifies the emerging future perspectives (Section 4.6).

### 4.1. Highlights, Detected Gaps, and Added Values

The narrative review of reviews (NRR) on digital twins (DTs) in oncology provides a comprehensive synthesis of the current research landscape, highlighting both the promises and the critical challenges associated with the integration of DTs into clinical oncology. The review systematically organizes the main thematic areas, identifies emerging opportunities, and examines the barriers to practical implementation—while also indirectly revealing important gaps and unmet needs that warrant further investigation. Below is a summary of the main insights.

*1.*  *Opportunities and Barriers* 

The NRR highlights several opportunities that DTs bring to oncology. DTs improve clinical decision-making by personalizing treatment based on patient-specific data. They also optimize therapy selection by predicting patient responses, thus enhancing treatment efficacy. Studies [21,23,27] show that DTs allow for better targeting of chemotherapy and radiotherapy, improving patient outcomes and minimizing side effects. Moreover, DTs aid in the early detection and prediction of cancer treatments, offering significant potential for personalized care.

However, the review also emphasizes key barriers. Data variability poses a challenge, as heterogeneous clinical data can reduce the accuracy of DTs. The validation of DT models in diverse clinical settings remains difficult due to the complexity of cancer patient populations. References [29,30] underlined that high-quality, standardized data are essential for accurate simulations. Moreover, computational demands are high, and real-time simulations require substantial resources, which may limit the widespread adoption of DTs.

*2.*  *Technical and Clinical Challenges* 

A significant barrier to DT implementation is the heterogeneity of cancer, making it difficult to model accurate, patient-specific representations. DTs must account for tumor variations both across patients and within tumors, adding complexity to model development. Studies [21,24,28] show that tumor heterogeneity complicates efforts to create universally applicable DTs, especially considering tumor mutations and microenvironment factors. Furthermore, clinical validation remains a major challenge. Ensuring that DTs align with actual patient outcomes is crucial for their integration into routine clinical practice.

*3.*  *Interdisciplinary Approaches and AI Integration* 

The NRR highlights the importance of interdisciplinary collaboration in advancing DTs. The convergence of artificial intelligence (AI), mechanistic modeling, and clinical expertise significantly enhances the predictive power of DTs, as well as their clinical utility. For example, studies such as [35,37] demonstrate how the integration of computational modeling with clinical biomarkers and real-world data enables more accurate simulations of tumor progression and therapy responses. These hybrid AI models represent a convergence of engineering, computer science, and oncology, allowing for nuanced simulations of complex biological phenomena.

Collaboration among computational scientists, biomedical engineers, and clinicians is evident in the development of theragnostic digital twins (TDTs), as discussed in [31]. These DTs personalize radiopharmaceutical therapies by combining advanced radiation physics, AI-driven dosimetry, and patient-specific clinical data. Similarly, in [23,24], interdisciplinary teams have combined imaging, pathology, and real-time simulations to refine diagnostic pathways and simulate personalized treatment responses, reflecting a strong collaboration between radiologists, oncologists, and data scientists.

Another clear instance is provided by [34], where optimal control theory and data assimilation—concepts rooted in applied mathematics and control engineering—are employed to refine clinical interventions. This highlights the integration of theoretical mathematical modeling with medical decision-making processes. Furthermore, the use of verification, validation, and uncertainty quantification (VVUQ) in [25] showcases how engineering rigor is critical to ensuring that DTs meet clinical safety and reliability standards.

Ultimately, interdisciplinary collaboration is not merely beneficial but foundational to the evolution of DTs in oncology. It ensures that these digital representations are not only technically robust but also clinically meaningful, enabling more precise, personalized, and safe treatment strategies.

*4.*  *Toward a Complementary Perspective: Future Directions, Ethics, and Market Readiness* 

An important dimension emerging from the NRR is the need to complement the current overview with forward-looking aspects, particularly concerning the projected growth of the field, ethical and legal implications, and concrete translational steps toward implementation. While the current body of literature provides a solid conceptual and technical foundation, there is growing recognition that the successful adoption of digital twins (DTs) in oncology also hinges on broader contextual factors.

Firstly, ethical and legal considerations are becoming increasingly central. The deployment of DTs involves the collection, integration, and real-time use of sensitive health data, including genomics, imaging, and behavioral information. This raises concerns related to patient privacy, data ownership, informed consent, and algorithmic transparency. As highlighted in the emerging literature, questions about accountability—especially in cases where DT-informed decisions impact clinical outcomes—remain largely unresolved. Furthermore, biases embedded in training datasets or simulation assumptions could perpetuate health disparities if not critically addressed. Thus, ethical-by-design frameworks and regulatory guidance will be crucial to ensure that DTs are not only technically accurate but also socially responsible and equitable.

Secondly, legal and regulatory frameworks are not yet fully adapted to the complexity and dynamic nature of DTs. The classification of these tools—as software medical devices, decision support systems, or research tools—varies across jurisdictions, which complicates the path to approval and widespread deployment. Existing norms around clinical trials, safety assessment, and software validation may need to be reinterpreted or expanded to encompass the evolving role of DTs in precision medicine.

Thirdly, to bridge the gap between research and clinical translation, it is essential to consider the market readiness of DT-based platforms. The review also needs to incorporate a mapping of available devices and platforms. This could involve tools for mechanistic modeling, AI-driven simulation, and patient-specific treatment planning. Their availability could signal a shift from research promise to practical utility and invite a reflection on how these technologies could be integrated into existing clinical workflows.

Moreover, insights from cutting-edge research—which go beyond the scope of the reviews covered in the NRR—could offer a glimpse into the next generation of DTs. Incorporating such emerging directions could allow for a more dynamic and forward-facing perspective, outlining where the field is headed and what obstacles remain on the horizon.

In this sense, the added value of complementing the NRR with ethical foresight, translational analysis, and state-of-the-art innovations lies in offering a more holistic view of how DTs can be responsibly and effectively scaled in oncology. It shifts the focus from “what exists” to “what is needed next,” opening a space for strategic reflection and future planning in both research and policy. The following discussion aims to fill this gap by integrating ethical foresight, regulatory analysis, and translational pathways, offering a more holistic view of how DTs can be responsibly and effectively scaled in oncology. It shifts the focus from “what exists” to “what is needed next”.

### 4.2. The Evolution of Digital Twins: Market Expansion and Oncological Impact

#### 4.2.1. Market Trends and Economic Forecasts for Digital Twins

The global digital twin market is experiencing substantial growth [38,39]. The market is projected to expand from USD 10.1 billion in 2023 to USD 110.1 billion by 2028, growing at a compound annual growth rate (CAGR) of 61.3%. This growth is driven by the increasing adoption of digital twins across various industries, including manufacturing, automotive, and healthcare. Similarly, Grand View Research estimates the market at USD 16.75 billion in 2023, forecasting a CAGR of 35.7% from 2024 to 2030, highlighting a broader trend toward growing investments and applications across various sectors.

#### 4.2.2. Digital Twins in Healthcare

In healthcare, digital twin technology is becoming a pivotal tool for personalizing treatments. By creating virtual replicas of patients and integrating clinical and physiological data, digital twins enable predictions of how patients will respond to specific treatments, optimizing care plans. Grand View Research projects the healthcare digital twin market to reach USD 902.59 million in 2024, with a robust growth trajectory, increasing at a 25.9% CAGR from 2025 to 2030 [40,41].

The adoption of digital twins in healthcare is particularly focused on improving clinical decision-making, simulating treatments for better precision, and enabling personalized care strategies. However, challenges such as data integration, data privacy, and computational demands remain significant barriers to full-scale implementation.

#### 4.2.3. Digital Twins in Oncology

Projections indicate significant growth in the digital twin market within healthcare, with an estimated USD 21.1 billion expected by 2028 [42]. Although specific data for oncology are not readily available, it is reasonable to assume that a substantial portion of this market will be attributed to oncology applications, given the increasing importance of these technologies in the field.

The European Organization for Research and Treatment of Cancer (EORTC) along with the National Cancer Institute (NCI) degli Stati Uniti e dall’American Association for Cancer Research (AACR) have emphasized and highlighted the relevance to the use of digital twins to simulate tumor behaviors and tailor therapies, aiming to optimize therapeutic responses and improve patient survival rates [43,44].

The major increasing application areas of digital twins in oncology (see Table 3, Table 4 and Table 5) include therefore the the following:

- **Treatment Optimization**: Predicting responses to chemotherapy, radiotherapy, and immunotherapy, while personalizing doses to reduce side effects.
- **Clinical Trial Simulations**: Using digital twins to simulate clinical trials, predicting patient responses, and optimizing trial design.
- **Therapeutic Decision Support**: Providing real-time data on tumor evolution and how tumors might respond to ongoing treatments, offering valuable support to oncologists.

These approaches aim to improve treatment effectiveness and reduce risks associated with traditional therapies.

### 4.3. Comparison and Contribution of Cutting-Edge Research

It is important, with reference to the directions of opportunities and challenges emerging in Table 5, to analyze how cutting-edge research (CER) is progressing and contributing into this direction. The following CER studies [45,46,47,48,49,50,51,52,53,54,55,56,57,58,59,60] were selected based on their focus on the opportunities and barriers reported in Table 5. The cutting-edge research (CER) in the field of digital twins (DTs) in oncology and precision medicine has been advancing rapidly, driven by interdisciplinary approaches and emerging technologies. This research, as highlighted in Table 6, demonstrates significant progress and introduces opportunities and challenges that are transforming the landscape of cancer treatment and care.

By analyzing the contribution of each study, it becomes clear how digital twins, integrated with artificial intelligence (AI), machine learning (ML), and other data sources, are reshaping clinical decision-making. These advancements enable highly personalized treatment strategies, improve diagnostic accuracy, and potentially reduce the cost and duration of clinical trials. However, these opportunities come with substantial challenges, including the integration of complex data, algorithm development, and addressing ethical, regulatory, and operational barriers.

The table “Contribution and Comparison to CER” is designed to showcase the relationship between cutting-edge studies in the context of integrating digital twin technology into oncology, specifically focusing on their additional contributions in relation to the insights and findings provided by the reviews in the NRR. The structure of the table is as follows:

1. **Reference:** 
  This column provides the citation of the study or review.

2. **Contribution:** 
  The Contribution column outlines the primary focus and key findings of each referenced study. It details how each study contributes to advancing digital twin technology’s application in precision oncology. This includes for example the integration of artificial intelligence (AI) and machine learning (ML) with DTs to personalize cancer treatment, improve diagnostic accuracy, and optimize therapeutic decisions. It also covers the operational challenges, such as the complexities in data technology, algorithm development, and integrating digital twin models into clinical workflows.

3. **Additional Insights:** 
  The Additional Insights column provides further reflections or novel findings that emerge from the study. It highlights aspects that are not commonly discussed in the literature or that contribute uniquely to the field. For example, in the study referenced, the use of AI/ML for generating synthetic data (digital twins) is mentioned as an innovative approach to expedite clinical trials, which adds an important dimension to the broader conversation of digital twins in oncology.

Together, the columns in this table offer a comprehensive look at how the integration of digital twin technology into oncology is advancing, while also drawing attention to additional elements that further enrich our understanding of the topic.

The integration of AI/ML technologies with digital twins (as explored in study [45]) presents a novel approach for accelerating clinical trials, providing synthetic data that can be used to simulate patient outcomes. Similarly, the study on theranostic digital twins [46] highlights a shift towards personalized radiotherapy, addressing not only technical challenges but also social, ethical, and regulatory issues associated with data sharing and patient consent.

Other studies, such as [47] on computational oncology for in silico clinical trials and [48] on cancer patient digital twins for immune response, further emphasize the potential for digital twins to enhance personalized cancer treatment by simulating patient-specific responses to therapies. Each of these studies highlights the growing importance of interdisciplinary collaboration, blending physics, AI, clinical practice, and computational biology to optimize patient care.

Despite the promising directions outlined in Table 6, there are significant challenges, particularly in terms of data management, system integration, and clinical adoption. As digital twins move from theoretical models to practical applications, overcoming these barriers will be essential for realizing their full potential in precision oncology. 

**Table 6:** Contribution and comparison to CER.

Reference	Contribution	Additional Insights
[45] Integration of AI/ML and Digital Twins in Precision Oncology	This study explores the convergence of AI/ML technologies with digital twins (DTs) to enhance precision oncology. It emphasizes the integration of multi-dimensional, multi-omic, spatial pathology, and radiomic data to enable personalized treatment strategies, improving diagnostic accuracy and therapeutic decisions. The study also highlights operational challenges like data technology, algorithm development, and data sharing in clinical workflows.	The application of AI/ML in generating synthetic data (digital twins) and its role in expediting clinical trials is a novel aspect not fully covered in previous studies.
[46] Theranostic Digital Twins for Precision Radiopharmaceutical Therapy	This research discusses the use of theranostic digital twins (TDTs) for personalizing radiopharmaceutical therapy (RPT). It integrates clinical, biomarker, image-based, and dosimetric data to optimize treatment, moving away from the one-size-fits-all approach. The study also addresses the social, ethical, and regulatory challenges in TDT implementation, such as data sharing, consent, and reimbursement.	Focuses specifically on theranostic applications and real-time treatment monitoring, providing additional insights into how DTs can personalize radiotherapy—an area not previously explored in-depth.
[47] Computational Oncology for In Silico Clinical Trials	This study explores the use of quantitative system pharmacology (QSP) models, virtual patients, and digital twins in predicting tumor responses to treatments, particularly for immuno-oncology therapies. The approach aims to reduce clinical trial time and costs.	The application of digital twins in simulating virtual patients for clinical trials, especially in immuno-oncology, offers a novel perspective not addressed in earlier studies.
[48] Cancer Patient Digital Twins for Immune Response	This work introduces cancer patient digital twins (CPDTs) for analyzing immune responses to metastasis. It utilizes multiscale mathematical models to simulate immune surveillance in cancer progression, highlighting how digital twins can dissect cancer complexity at the individual level.	The study provides new insights into immune responses and metastasis, incorporating computational models to analyze immune system interactions with cancer—extending digital twins’ application beyond tumor dynamics.
[49] Prostate Cancer Digital Twin Framework	This study presents a framework for creating personalized digital twins in prostate cancer (PCa). It integrates clinical MRI data and simulates tumor growth using methods like the Finite Element Method (FEM), along with patient-specific biomarkers like PSA levels. The study also employs a multi-objective optimization process for model adjustments.	This case study offers a concrete example of digital twin creation for prostate cancer, focusing on MRI data integration and computational simulations to predict tumor growth and PSA dynamics, providing valuable insights for DT development in specific cancer types.
[50] Generalizing Digital Twins and Complexity Data Science	This study broadens the concept of digital twins, positioning them as part of a larger field that connects complexity science with data science. The paper introduces the term “complexity data science” and explores the historical, theoretical, and future implications of this approach.	The integration of complexity science with digital twins opens new interdisciplinary pathways, suggesting that digital twins can be a cross-domain tool for scientific exploration beyond traditional applications.
[51] Digital Twins in Neuroscience: Modeling Brain Functions and Pathology	The study applies digital twin technology to model brain tumors, offering insights into how brain structure and function can be impacted by tumors. This method is directly translatable to oncology, particularly in understanding tumor progression, treatment responses, and the effects of therapies on brain function, which is essential for personalized care in brain tumor patients.	The interdisciplinary approach combines neuroscience and digital twin technology, offering valuable insights into tumor dynamics and treatment planning in oncology. These insights could be extended beyond brain tumors to other areas of cancer care, supporting a more personalized, data-driven approach to treatment in oncology.
[52] Optimizing Fentanyl Dosing with Physics-Based Digital Twins	Focuses on using physics-based digital twins (PBDTs) to personalize fentanyl dosing for cancer patients, incorporating clinical and physiological parameters. The study outlines a clinical protocol for validating PBDT-guided dosing and emphasizes therapeutic drug monitoring.	This approach applies digital twins in pain management, particularly in oncology, offering a personalized and clinically relevant strategy to optimize opioid dosing and minimize risks for advanced cancer patients.
[53] Promoting FAIR Data Sharing in Biomedical Research	Presents a digital twin-based system to facilitate FAIR sharing of sensitive biomedical data. The system allows non-IT-affine users to generate synthetic data and enrich existing datasets, promoting open science and reproducibility in research.	By making data sharing easier for non-technical users and fostering collaboration, this work advances the integration of digital twins into data management systems, significantly enhancing the accessibility and utility of biomedical data.
[54] Predicting Treatment Outcomes with Mathematical Models and Digital Twins	Investigates mathematical models and digital twins to predict treatment outcomes for lung cancer patients undergoing immune checkpoint inhibitor therapy. The study integrates a delayed response model to predict disease progression with high accuracy.	This research shows how digital twins, in combination with mathematical models, can provide clinicians with actionable insights, improving treatment decision-making in oncology and helping predict patient outcomes with precision.
[55] Machine Learning and Radiomics in Non-Small Cell Lung Cancer	Explores the value of healthy organ data alongside tumor tissue for survival prediction in non-small cell lung cancer using PET/CT images and radiomics.	PET and CT Organomics significantly enhance survival prediction models. This approach is key for developing digital twins in oncology, improving patient outcomes.
[56] Digital Twins for OvCa Patients and Caregivers	Creates digital twins of OvCa patients and caregivers, using data from forums, interviews, expert input, and clinical notes to represent diverse cancer trajectories and needs.	The digital twins enable AI model training in scenarios with limited user data, offering a promising tool for personalized healthcare and decision support systems in cancer care.
[57] Cell State Transition Assessment for Breast Cancer	Applies the cSTAR approach to analyze phosphoproteomic data from breast cancer cell lines, identifying key signaling nodes and causal connections within core control networks.	cSTAR models offer a mechanistic understanding of cancer subtypes and suggest strategies for normalizing signaling networks, potentially offering therapeutic avenues for breast cancer.
[58] Physics-Based Digital Twin for Transdermal Fentanyl Delivery	Develops a physics-based digital twin to optimize fentanyl delivery through transdermal patches, considering factors like skin characteristics, temperature, and activity levels.	Temperature regulation and personalized patch placement improve fentanyl delivery consistency, reducing variability in pain management outcomes, offering a tool for clinical decision-making.
[59] Simplifying Patient-Specific CFD Simulations for Hepatocellular Carcinoma Treatment	Evaluates simplification strategies for CFD simulations in hepatocellular carcinoma treatment, reducing computational time while maintaining accuracy.	Grid coarsening effectively reduces computational time by 45%, maintaining accuracy, and improving the efficiency of patient-specific simulations for personalized cancer treatment.
[60] Applications of Digital Twins in Laboratory Medicine	Explores digital twins in laboratory medicine, focusing on personalized treatment plans, biological variation data, and AI-driven lab test interpretation.	Digital twins combined with AI and synthetic data can revolutionize laboratory medicine, improving personalized diagnostics and treatment planning, and enhancing healthcare precision.

### 4.4. Integrating Digital Twins: Navigating Ethical and Legal Complexities

A strategic aspect for the successful and effective integration of DTs is the need to address ethical and legal considerations. These aspects are particularly complex and far-reaching, making it essential to conduct a dedicated, in-depth analysis. Such an analysis should explore the different strategies, regulatory frameworks, and compliance actions required at both national and international levels.

Furthermore, a comprehensive approach should consider existing guidelines and protocol documents that define best practices for the adoption and governance of DTs. These frameworks play a crucial role in ensuring that digital twins are developed and implemented in a way that is transparent, fair, and aligned with regulatory requirements across different jurisdictions.

Additionally, many of the legal and ethical challenges surrounding DTs are closely tied to the rapid advancements in artificial intelligence (AI). As we have seen, AI and DTs are strongly interconnected, with AI often serving as the driving force behind the ability of digital twins to analyze data, simulate scenarios, and optimize real-world systems. However, this interconnection also brings new challenges related to data privacy, security, accountability, and decision-making autonomy.

As AI continues to evolve, regulations and ethical guidelines must adapt to ensure that digital twin technologies are developed responsibly. This includes addressing concerns such as bias in AI-driven predictions, the ownership and use of sensitive data, liability in automated decision-making, and the potential societal impact of large-scale digital simulations.

Ultimately, achieving a real and sustainable integration of digital twins will require a multidisciplinary effort, bringing together technologists, legal experts, policymakers, and ethicists to create a framework that balances innovation with accountability and public trust.

Several studies have explored the ethical, legal, and governance challenges associated with digital twins in different domains, selected by narrowing down the research keys with this focus [61,62,63,64].

Advances in digital medicine now make it possible to use digital twin systems (DTS), which combine extensive patient monitoring through multiple sensors with personalized adaptation of patient care via software [9]. With the artificial pancreas system already operational in children with type 1 diabetes, new DTS could be developed for real-time monitoring and management of children with chronic diseases. However, pediatric care presents specific challenges, including ethical concerns related to the child’s role in decision-making, data collection difficulties, and the need to maintain human interaction between patients and healthcare providers. These considerations are crucial in ensuring that DTS applications in pediatrics do not contribute to an excessive surveillance society or unintended environmental consequences.

The role of digital twins in sustainable development has been highlighted in the European Union (EU) context. By leveraging big data and artificial intelligence, DTs can simulate states, reactions, and potential developments of physical systems, forming a robust foundation for data-driven policy decisions [61]. A key objective of DTs is to support the implementation of the EU’s Green Deal, aligning with internationally binding climate and environmental targets. However, achieving this goal requires a comprehensive, high-quality database supported by a legal framework ensuring open and fair access to data. While EU digital law has made progress in this regard, further steps are needed to link digital twins to legally binding sustainability objectives.

Digital Health Technologies (DHTs), including digital twins, raise significant ethical, legal, and societal implications (ELSI). Current regulatory frameworks often lag behind technological advancements, especially concerning medical devices [63]. Digital twins represent the next evolution of DHTs and offer an opportunity to address these challenges proactively. By drawing comparisons with machine learning and personalized medicine, researchers are mapping ethical dilemmas such as patient autonomy, data security, and liability in clinical decision-making. Addressing these challenges is essential to ensure that digital twins enhance medical practice without compromising fundamental rights and ethical principles.

In the context of oncology, digital twins offer promising applications in personalized treatment and real-world data integration. One key challenge in cancer research is the limited availability of robust clinical trial data. The COVID-19 pandemic underscored this issue by highlighting the ethical and methodological complexities of expanded access (EA) programs. While these programs provide compassionate use of investigational therapies, they lack the rigor of randomized controlled trials (RCTs). Digital twins can help bridge this gap by leveraging real-world data to derive causal inferences and improve clinical decision-making [64]. This approach can enhance oncology research by ensuring that data from EA programs contribute meaningfully to the development and approval of new treatments.

Finally, the legal liability of digital twins in healthcare remains a critical issue. As highlighted in [62], the integration of DTs into medical practice raises questions about accountability in cases of errors or misdiagnoses. Determining liability in AI-assisted medical decisions requires a clear legal framework that defines the roles and responsibilities of developers, healthcare providers, and regulatory bodies. Addressing these concerns is essential for fostering trust in digital twin technologies and ensuring their ethical and legal adoption in medicine.

Overall, the European experience [61] serves as a valuable case study in the integration of digital twins, demonstrating both their potential benefits and the challenges that must be addressed. By drawing from EU regulatory efforts, particularly in data governance and sustainability, policymakers and researchers can develop a roadmap for responsible digital twin implementation worldwide. This includes ensuring that these technologies contribute positively to healthcare, oncology research, and environmental sustainability while upholding ethical and legal standards.

### 4.5. Core vs. Supportive Technologies in Digital Twins: Available Platforms and Their Roles in Oncology

The following provides an example of commercial devices and platforms that are leveraging digital twin technologies directly or indirectly in oncology. While this list is by no means exhaustive, neither is our aim to identify the “best of the bunch”; rather, we aim to highlight key examples that demonstrate the diverse applications and potential of digital twin technologies in advancing personalized cancer care. It offers a snapshot of how various tools contribute to the evolving field of personalized cancer treatment. Some platforms represent core technologies that directly build and maintain digital twin models for patients, acting as the foundational element in the development of personalized healthcare. These core platforms are responsible for constructing detailed, dynamic virtual replicas of patients, integrating a wide array of clinical data—such as genomics, imaging, and physiological measurements—into one cohesive model. This allows clinicians to simulate how a patient’s body or a specific disease might respond to various treatments, providing personalized predictions to guide decision-making in real-time.

On the other hand, other platforms serve as supportive technologies, which play an equally vital role in enriching and enhancing the overall digital twin framework. While they do not directly create or update the patient’s digital twin, these platforms provide critical functionality that improves the accuracy, usability, and clinical applicability of digital twins. These supportive technologies often focus on processing vast datasets (such as multi-omic data or medical imaging), analyzing complex patterns, and offering valuable insights or therapeutic recommendations. By augmenting the core digital twin models, they help bridge gaps in data interpretation, simplify visual representation, and refine treatment predictions, thus enhancing the overall clinical utility of the digital twin framework.

In essence, while core technologies are central to the actual creation and operation of digital twins, supportive technologies provide essential tools that augment, enhance, and streamline the workflows associated with these patient-specific models. Together, both types of platforms work synergistically to advance the field of personalized medicine and improve the quality of patient care.

Table 7 offers a representative selection of commercial platforms currently applied in oncology-related digital twin solutions.

**Table 7:** Devices and platforms for digital twin applications in oncology.

Reference	Device/Platform Name	Function in Oncology Digital Twin	Category
[65]	ModCell™	Mechanistic modeling system for predicting tumor response to specific therapies, integrating omic data and in silico simulations.	Core
[66]	Cytocast™ Digital Twin	Platform that creates a digital twin of the patient using multi-omic and clinical data to simulate drug efficacy and side effects.	Core
[67]	Quibim QP-Insights	Cloud platform for the management and quantitative analysis of multi-omic data, supporting precision medicine and oncology research.	Support
[68]	CERTAINTY Virtual Twin	European project developing a virtual twin for supporting personalized cancer treatment, focusing on advanced therapies like CAR-T cells.	Support
[69]	Q Bio Gemini	Platform for creating a digital twin of the entire human body, integrating genetic, biochemical, imaging data, and more for health monitoring.	Support
[70]	IBM Watson for Oncology	AI platform for analyzing oncology data, providing therapeutic recommendations based on scientific evidence.	Support

The table, “Devices and Platforms for Digital Twin Applications in oncology”, is designed to present key devices or platforms used in the development and application of digital twin technology in oncology. It organizes information based on the function of each device or platform within the context of oncology digital twins and categorizes them according to their role.

The following is a breakdown of the columns:

- **Reference**: This column cites the source of the device or platform, providing a reference for further exploration.
- **Device/Platform Name**: The specific name of the technology, system, or platform that is being utilized for oncology digital twin development.
- **Function in Oncology Digital Twin**: A brief description of how each device or platform contributes to the use of digital twins in oncology, focusing on its core functionalities and how it enhances personalized cancer treatment, tumor modeling, or therapy simulation.
- **Category**: This column categorizes the device or platform, for instance, whether it is considered a core or auxiliary component (supportive technology) in oncology digital twin systems.

The goal is to provide a clear overview of the tools and platforms that facilitate the integration of digital twin technologies into oncology, outlining their roles and categorizing their functions.

Let us take a closer look at the platforms in the table:

***Core Technologies***

These platforms are at the center of the digital twin concept, creating a dynamic, patient-specific model that simulates responses to various treatments. While core technologies are often associated with oncology in this context, it is important to note that they are not oncology-exclusive. These platforms are designed to support digital twin applications across various medical domains, with oncology being one of the primary areas of focus for integration.

- **ModCell™**: This platform stands as an example of how mechanistic modeling can predict tumor responses to therapies. By integrating omic data and performing in silico simulations, ModCell™ enables the construction of a highly personalized digital twin, essential for exploring how a particular tumor might behave under different treatments. This predictive capability can significantly improve decision-making processes in oncology, making it a core tool in the development of precision therapies. Though ModCell™ is often applied in oncology, it can be adapted to other clinical areas that require predictive modeling of disease mechanisms.
- **Cytocast™ Digital Twin**: Similarly to ModCell™, Cytocast™ leverages multi-omic and clinical data to create a digital twin of the patient. Its strength lies in simulating not just the tumor’s behavior but also the potential side effects of treatments. By providing a more holistic view of treatment outcomes, Cytocast™ helps doctors tailor therapy choices, balancing efficacy and minimizing risks for patients. While Cytocast™ is especially valuable in oncology, its modeling capabilities are applicable in other therapeutic areas where understanding complex treatment responses is critical.

***Supportive Technologies***

These platforms play an essential role in enriching and supporting the digital twin framework, whether through data analysis, visual modeling, or therapeutic recommendations. Although they do not directly build the digital twin models, they provide critical components that enhance the functionality and precision of core systems.

- **Quibim QP-Insights**: While not a digital twin in itself, Quibim QP-Insights provides valuable data processing capabilities, helping oncology teams manage and analyze multi-omic data. By extracting relevant insights, it enhances the foundation on which a digital twin can be built, improving the precision and predictive power of models. This tool is particularly useful in oncology, where large datasets need to be analyzed to support personalized treatment plans.
- **CERTAINTY Virtual Twin**: Although this platform is part of an ongoing European research project, it is an example of how virtual twin technology is being applied to personalized cancer treatment. It focuses on advanced therapies, like CAR-T cell treatments, providing simulations that inform both clinical trials and patient-specific treatment planning. Although it is still in the development phase, its potential to support digital twins in oncology is immense. CERTAINTY is not yet a fully realized product but shows significant promise in advancing virtual twin applications for oncology and beyond.
- **Q Bio Gemini**: Though Q Bio Gemini is primarily focused on whole-body health monitoring, its ability to create a comprehensive digital twin of a patient that integrates genetic, biochemical, and imaging data makes it a valuable resource in the oncology space. While it is not exclusively cancer-focused, the data it provides can be crucial in understanding how cancer develops and progresses, supporting digital twin models for personalized care. Q Bio Gemini’s wide-ranging health data make it applicable to a variety of clinical fields, not just oncology.
- **IBM Watson for Oncology**: Powered by AI, IBM Watson for Oncology analyzes vast amounts of oncology data to provide actionable insights and therapeutic recommendations. While not a traditional digital twin platform, its AI-driven support aids clinicians in making informed decisions, enhancing the digital twin models by feeding them with evidence-based, up-to-date treatment strategies. Watson’s ability to process and interpret large datasets is a key supportive feature for personalized cancer treatment.

This collection of technologies showcases the diversity of approaches being used to integrate digital twin technology into cancer treatment. By using both core and supportive technologies, healthcare professionals can better understand the complexity of cancer and its response to treatments, ultimately advancing the personalization of oncology care. Although the core platforms are not exclusive to oncology, their integration with cancer-specific models and data enhances their value in precision medicine. 

### 4.6. Future Directions

The long-term potential of DTs in oncology focuses on improving personalized treatment strategies. As the technology matures, real-time data assimilation and more accurate simulations will lead to more effective treatments. Addressing challenges like data integration, ethical and privacy concerns, and regulatory approval will be essential for the future of DTs. The review emphasizes that scalability remains a key challenge, as the computational resources needed for DT simulations may not be available in all clinical settings.

### 4.7. Limitations

This narrative review of reviews on digital twins (DTs) in oncology follows a structured methodology designed to provide a comprehensive understanding of the current literature. While several limitations exist, they also present valuable opportunities for further research and development.

One consideration is the exclusion of conference proceedings, preprints, and gray literature. While this review emphasizes peer-reviewed publications to ensure methodological rigor, the integration of these additional sources in future research could offer a more dynamic and up-to-date perspective on digital twin technology. This is especially relevant in rapidly advancing areas like AI-driven simulations and real-time treatment response modeling, where emerging trends and innovative pilot studies may offer novel insights.

Another consideration is the focus on English-language literature. While this helps to enhance the broad applicability of findings, expanding research to include multilingual studies could uncover region-specific insights, localized best practices, and culturally adapted strategies that might influence the adoption and effectiveness of digital twin technologies in oncology. Future studies could benefit from cross-cultural comparisons to capture a wider range of perspectives and ensure that digital twins are integrated in diverse healthcare contexts.

This review primarily synthesizes previously published studies, which is valuable for drawing overarching conclusions. However, incorporating more primary research, including qualitative and mixed-methods studies, could provide a richer understanding of the real-world application of digital twins. This would allow for a deeper exploration of patient experiences, healthcare provider perspectives, and the practical challenges of implementing these technologies in oncology settings.

The variability across reviewed studies in terms of methodologies, patient populations, and evaluation metrics reflects the rapidly evolving nature of digital twin technology. Rather than being seen as a limitation, this diversity underscores the importance of developing standardized frameworks and benchmarks. Such frameworks would facilitate clearer comparisons and more robust assessments of digital twin effectiveness, usability, and ethical considerations, ultimately enabling better integration into oncology practice.

While this review focuses on clinical applications, there are broader societal, ethical, and psychological dimensions of digital twin technology that merit further attention. Future studies could investigate the long-term impact of digital twins on healthcare providers, patient trust, and ethical issues surrounding data privacy, decision-making, and AI-driven treatment planning.

To address these areas and build on current knowledge, future research should include the following:

- Expand Data Sources: Integrate conference proceedings, preprints, and gray literature to capture emerging trends and novel applications.
- Enhance Cross-Cultural Insights: Conduct studies across different linguistic and cultural contexts to better understand region-specific adaptations and challenges.
- Integrate Primary Research: Complement reviews with qualitative and mixed-methods studies to capture real-world implementation challenges and patient experiences.
- Develop Standardized Evaluation Frameworks: Establish universal benchmarks for assessing digital twin effectiveness, usability, and ethical compliance.
- Explore Long-Term Social and Psychological Effects: Investigate the impact of digital twins on healthcare providers, patient trust, and the broader ethical landscape.
- Promote Interdisciplinary Collaboration: Foster collaboration among oncologists, engineers, ethicists, and policymakers to ensure responsible and impactful digital twin development.

By addressing these directions for future research, the field can continue to advance, leading to the responsible and effective integration of digital twins in oncology. This will ultimately enhance personalized cancer care and improve patient outcomes in the future.

## 5. Conclusions

A narrative review of reviews was performed, highlighting that digital twin (DT) technology, when integrated with artificial intelligence (AI) and machine learning (ML), has the potential to reshape oncology care. The synthesis of existing studies emphasizes how the integration of these advanced technologies can significantly improve personalized cancer treatment. By creating dynamic, virtual replicas of patients, DTs enable clinicians to simulate disease progression, treatment responses, and therapeutic outcomes, offering a tailored and more precise approach to oncology.

This review reveals that the application of DT technology in oncology provides several key advantages, including improved decision-making processes, enhanced treatment planning, and the possibility for real-time adjustments to therapy. It also highlights the innovative ways in which DTs are being utilized to optimize clinical trials, enhance drug development, and personalize radiotherapy and chemotherapy regimens.

However, the review also identifies challenges in the widespread implementation of DTs, such as data integration issues, the complexity of biological modeling, and the need for robust computational resources. Additionally, while DTs show promise in improving treatment strategies, the clinical validation of these models remains a significant barrier, requiring comprehensive efforts to ensure their accuracy and effectiveness in real-world clinical settings.

A strategic aspect for the successful and effective integration of digital twins (DTs) is the need to address ethical and legal considerations. These aspects are particularly complex and far-reaching, making it essential to conduct a dedicated, in-depth analysis. Such an analysis should explore the different strategies, regulatory frameworks, and compliance actions required at both national and international levels. Furthermore, many of the legal and ethical challenges surrounding DTs are closely tied to the rapid advancements in AI. As AI and DTs are strongly interconnected, this integration brings forth concerns related to data privacy, security, accountability, and decision-making autonomy.

As AI continues to evolve, regulations and ethical guidelines must adapt to ensure that digital twin technologies are developed responsibly. This includes addressing issues such as bias in AI-driven predictions, the ownership and use of sensitive data, liability in automated decision-making, and the societal impact of large-scale digital simulations. Ultimately, achieving a real and sustainable integration of digital twins will require a multidisciplinary effort, bringing together technologists, legal experts, policymakers, and ethicists to create a framework that balances innovation with accountability and public trust.

In this context, the European Union (EU) experience offers valuable lessons. The EU has made significant strides in digital law and data governance, providing a framework that ensures the responsible use of emerging technologies like digital twins. EU regulatory efforts emphasize the importance of transparency, fairness, and alignment with sustainability goals, such as those set out in the EU Green Deal. While the integration of digital twins in healthcare presents substantial promise, the EU’s approach to regulation also demonstrates the need for robust legal frameworks and collaborative efforts at the international level. This experience can serve as a model for other regions looking to integrate DTs in oncology and other medical fields, ensuring that they are used ethically and effectively to improve patient outcomes.

Looking forward, as the market for digital twins in healthcare continues to grow, it is crucial to remain vigilant in addressing challenges related to data quality, interoperability, and computational resources. With ongoing advancements in research, technology, and regulation, there is significant potential for digital twins to reshape oncology, offering a future where cancer treatment is more personalized, efficient, and effective. However, careful attention to ethical, legal, and operational considerations will be key to ensuring that these innovations are deployed in a way that benefits patients while maintaining public trust and safety.

## Figures and Tables

**Table 1:** Comparative definitions of digital twin technology.

Source	Definition	Emphasis
Gartner (2020) [1]	“A virtual representation of a real-world entity or system that uses real-time data to simulate behaviors and enhance decision-making.”	*Dynamic real-time data integration, behavior modeling, and decision support optimization.* This definition emphasizes the importance of integrating real-time data from the physical world into the digital model, enabling the simulation of behaviors that can enhance the decision-making process in dynamic environments. It highlights the application of digital twin (DT) technology as a decision support tool.
Digital Twin Consortium [2]	“An accurate virtual representation of an object, system, or process that continuously updates with real data to support monitoring, analysis, and optimization.”	*Real-time continuous data synchronization, high model accuracy, and data-driven decision-making for process optimization.* This source highlights continuous real-time updates to ensure the digital twin remains highly accurate over time. It underscores the role of the digital twin in supporting data analytics and optimization to improve processes and systems within industries.
NASA (2012) [3]	“A digital model of a physical system that integrates data, simulations, and analytics to understand, predict, and optimize its operation.”	*Comprehensive integration of analytics, predictive modeling, and operational optimization through simulations.* NASA’s definition focuses on the ability of digital twins to integrate a variety of data types (including simulations and analytics) to predict behaviors and optimize operations of physical systems. It emphasizes the utility of digital twins for long-term operational efficiency and predictive maintenance.
ISO 23247-1:2021 [4]	“A set of interconnected digital models that replicate the characteristics of a physical entity, enabling bidirectional interactions between the real and virtual worlds.”	*Interconnected, multi-dimensional digital models with bidirectional interaction for seamless integration between virtual and physical environments.* ISO’s definition emphasizes the interconnectedness of digital models that replicate real-world entities. The concept of bidirectional interaction stands out, ensuring that changes in the physical world are reflected in the digital twin and vice versa, which is essential for continuous monitoring and optimization. The alignment with Industry 4.0 highlights the advancement towards smart, automated industrial processes.

**Table 2:** Key applications of digital twins in oncology.

Domain	DT Applications	Technologies/Data Integrated	Purpose
Imaging (MRI, PAI)	Real-time tumor modeling; monitoring of tumor microenvironment changes	MRI, photoacoustic imaging, ultrasound, CT scans	Enhanced tumor visualization; dynamic tracking of tumor evolution
Diagnosis	Predictive modeling of cancer onset and progression; risk stratification	Genomics, liquid biopsies, histopathology images	Early detection; accurate staging and prognosis
Chemotherapy	Simulation of pharmacokinetics and pharmacodynamics; optimization of drug regimens	Pharmacogenomics, treatment response data	Predict therapeutic efficacy; reduce toxicity
Radiotherapy	Personalized radiation dose planning; adaptation during treatment	CT, MRI, radiomics data	Maximize tumor control; minimize damage to healthy tissue
Photodynamic Therapy (PDT)	Optimization of light dosimetry and photosensitizer administration	Optical imaging; tissue oxygenation measurements	Improve therapeutic outcomes; reduce side effects
Photothermal Therapy (PTT)	Simulation of heat distribution; optimization of energy delivery	Thermal imaging; nanoparticle distribution models	Precision ablation of tumors; preservation of healthy tissue

**Table 3:** Synthesis of digital twin applications and innovative approaches in oncology.

Reference	Brief Description	Focus on Oncology	Innovative Approach
[21]	Scoping review mapping externally validated machine learning (ML)-based models in cancer care, assessing their performance, clinical utility, and model relationships.	Although the focus is on machine learning, it contributes to the integration of ML with digital twin concepts in oncology by improving clinical decision-making and cancer patient care.	Integration of ML with digital twin concepts for oncology decision support.
[22]	Discusses digital twins (DTs) for personalized treatments in oncology, addressing challenges like data integration and real-time simulation for patient-specific treatments.	Direct focus on oncology with applications in cancer treatment, disease prevention, and personalized therapies.	Digital twins (DTs) used to simulate disease and treatment pathways, enabling more personalized cancer care.
[23]	Review on the integration of AI and DTs in oncology, focusing on the usage of imaging, pathology, radiotherapy, and genomics.	Oncological applications including imaging and genomics-driven therapies powered by DTs.	AI and DTs work together to enable precision medicine, improving diagnostic accuracy and treatment effectiveness in oncology.
[24]	Exploration of digital twins (DTs) for improving healthcare, particularly in oncology and neurology, including challenges in data integration.	Oncology-specific applications, such as diagnostic simulations and predictive modeling for cancer progression thanks to the advancing medical knowledge in neuroscience.	Demonstrates the potential of digital twin technology to model patient-specific oncology scenarios, allowing for tailored treatment plans, while simultaneously highlighting the importance of neuroscience in understanding complex tumor behaviors and brain–cancer interactions.
[25]	Focus on the role of verification, validation, and uncertainty quantification (VVUQ) for digital twins, with applications in oncology.	Direct focus on the use of DTs in oncology, particularly in ensuring the safety and efficacy of patient simulations.	VVUQ framework helps ensure the reliability and clinical utility of DTs in oncology.
[26]	Scoping review of digital twins (DTHs) and virtual twins (VTHs) in oncology, examining technical solutions, challenges, and credibility.	Broad coverage of oncology types including breast, lung, and colorectal cancers, focusing on diagnosis, therapy, and monitoring.	Digital twins in oncology for personalized diagnosis and treatment simulations, helping in therapy optimization.
[27]	Discusses advancements in computational methodologies for precision oncology, with an emphasis on cancer digital twins (DTs) for patient-specific decision-making.	Focuses on cancer digital twins for precision oncology, integrating patient-specific data and mathematical models for improved clinical decisions.	Development of cancer digital twins using agent-based modeling and integration of tumor microenvironment simulations.
[28]	Outlines the potential of generative digital twins (GDTs) in biomedical research, focusing on the creation of high-fidelity virtual replicas for oncology applications.	Applies generative digital twins in oncology for personalized diagnostics and treatments.	Use of generative AI to create spatially resolved digital representations of tumors and biological entities.
[29]	Reviews the use of digital twins in radiotherapy, focusing on personalized cancer treatments guided by mechanistic patient-specific simulations.	Oncology focus on personalized radiotherapy and the use of digital twins to guide treatment adaptations based on patient-specific data.	Personalized digital twins in radiotherapy to improve tumor control while minimizing damage to healthy tissues.
[30]	Discusses the challenges of generating virtual patient populations and digital twins for immuno-oncology, with a focus on personalized models for specific clinical settings.	Immuno-oncology applications, focusing on creating digital twins for cancer treatment personalization.	Development of digital twins for immuno-oncology research, with study-specific models tailored to clinical needs.
[31]	Proposes theranostic digital twins (TDTs) to personalize radiopharmaceutical therapy (RPT) based on patient-specific data to optimize treatment and minimize toxicity.	Focus on radiopharmaceutical therapy in oncology, using TDTs to optimize radiation dose and minimize side effects.	Theranostic digital twins (TDTs) that personalize cancer treatment based on real patient data, improving precision and reducing risks.
[32]	Explores the potential of mechanistic learning (ML) combining mathematical modeling and machine learning in oncology, with applications in tumor prediction and response modeling.	Focus on oncology research, utilizing mechanistic learning for tumor response prediction and time-to-event modeling.	Synergistic use of mechanistic mathematical modeling and machine learning, including the integration of digital twins in oncology.
[33]	Provides an overview of medical digital twins, highlighting their application in oncology and cardiology, and discusses major challenges such as data integration and privacy.	Focuses on digital twins in oncology for personalized treatments and diagnostics.	Emphasizes the use of digital twins to personalize medicine and improve patient outcomes, while addressing challenges in data integration.
[34]	Describes a computational framework for personalized clinical trials using digital twins, mathematical modeling, and data assimilation.	Applies digital twins in oncology to simulate patient-specific interventions and optimize treatment outcomes.	Use of digital twins in personalized clinical trials, applying optimal control theory and data assimilation to refine predictions and interventions.
[35]	Discusses model-informed precision medicine using AI and ML, focusing on the integration of digital twins in drug development, particularly in oncology.	Focuses on oncology and immuno-oncology, using digital twins to personalize treatments based on multi-dimensional biomarker data.	Use of AI/ML to integrate real-world and -omics data, enabling model-informed precision medicine with digital twins for better treatment outcomes.
[36]	Reviews the potential of digital twins in oncology, specifically through image-guided, mechanism-based, tissue-scale mathematical modeling.	Focuses on oncology, particularly using digital twins for tumor dynamics and patient-specific care.	Development of image-guided, mechanism-based digital twins to model tumor behavior and personalize cancer treatment.
[37]	Discusses the application of AI and digital twins in precision medicine for inflammatory bowel disease (IBD) and their potential use in oncology.	While focused on IBD, the review highlights the potential of digital twins in oncology, particularly in predictive modeling and precision dosing.	Combines mechanistic modeling and AI to create digital twins for personalized treatments in IBD and oncology.

**Table 4:** Study categorization.

Category	Key Topics	Key References
1. Foundations and Frameworks for Digital Twins in Oncology	Computational frameworks for precision oncology using digital twins (DTs), agent-based modeling for simulating cancer dynamics, patient-specific data for personalized treatments, data assimilation to continuously update models	[27,33,34]
2. Innovative Applications of Digital Twins in Oncology	Application of DTs in radiotherapy for personalized treatment planning, tumor control, minimizing normal tissue damage using patient-specific DT models, development of theranostic digital twins (TDTs) to optimize radiopharmaceutical therapies and minimize toxicity	[29,31,36]
3. Technological Integration: Data, Machine Learning, and Modeling	Integration of AI and ML techniques with DTs to enhance precision medicine in oncology, enabling the use of real-world data, and improving drug development and treatment strategies, hybrid models combining mechanistic and AI-driven approaches for creating DTs, model-informed precision medicine using DTs for optimized treatment planning	[21,35,37]
4. Challenges in Digital Twin Development	Key challenges in creating spatially resolved generative DTs for oncology, such as integrating multi-modal data (e.g., imaging, genomics) and biological complexity, generating immuno-oncology-specific DTs tailored to clinical settings, the difficulty of overcoming cancer heterogeneity using DTs	[26,28,30]
5. Clinical Utility and Impact of Digital Twins in Oncology	The potential of DTs to improve clinical decision-making, assist in patient-specific treatment planning, and enhance treatment outcomes by simulating real-time patient responses, mechanistic learning models used with DTs for optimizing treatment protocols in oncology	[24,32]
6. Future Directions and Long-term Goals for Digital Twins in Oncology	Long-term potential of DTs in oncology, focusing on their ability to improve personalized treatments, address challenges like data integration, privacy concerns, and enhance the understanding of tumor dynamics for more effective treatment planning	[27,33]
7. Cross-Disciplinary Approaches	Cross-disciplinary applications of DTs in oncology and other fields (e.g., IBD), the integration of AI-driven hybrid models for creating and utilizing DTs to enhance therapeutic responses, collaborative approaches to data analysis for DTs across multiple domains	[35,37]
8. Personalized Trials and Patient-Centered Models	Development of computational frameworks for personalized clinical trials using DTs, real-time data assimilation to continuously refine patient models, optimizing trial designs by integrating patient-specific data with DTs to simulate treatment outcomes	[34]

**Table 5:** Opportunities and challenges in oncology and digital twin integration.

Study	Opportunities	Barriers
[21]	**Enhancement of clinical decision-making**: DTs can integrate machine learning models into oncology practice to provide personalized treatment decisions based on patient data. **Improved treatment strategies**: DTs help optimize therapy selection by predicting the response to different cancer treatments.	**Data variability**: Challenges in dealing with heterogeneous clinical data, which can affect the reliability of the DTs. **Clinical validation**: Difficulty in validating DT models in diverse, real-world clinical settings due to the complexity of the cancer patient population.
[22]	**Personalized treatment plans:** DTs can predict and simulate patient-specific responses, allowing oncologists to fine-tune therapies for each patient. **Early diagnosis and intervention**: The use of predictive models to detect cancer and identify the most effective treatment modalities early on.	**Complexity of modeling:** Difficulty in developing accurate, patient-specific DTs due to the complexity and variability of biological data. **Scalability:** Issues with applying predictive models across a large and diverse patient base in real-world clinical settings.
[23]	**Optimization of therapy regimens**: DTs can simulate and predict optimal chemotherapy or radiotherapy protocols tailored to individual patients, increasing treatment efficacy. **Enhancement of treatment outcomes**: Personalized models can improve patient survival rates by tailoring therapies to specific tumor dynamics.	**Computational demand**: Real-time simulations for patient-specific treatment planning can be computationally expensive and resource-intensive. **Data limitations:** Insufficient or low-quality patient data limits the ability of DTs to accurately simulate patient-specific responses.
[24]	**Revolutionizing oncology care**: Digital health twins (DHTs) can integrate large-scale data, including genetic and environmental factors, to offer truly personalized care. **AI integration:** Using AI to enhance the predictive accuracy of DTs, improving treatment and monitoring in oncology.	**Infrastructure challenges:** Existing healthcare systems may lack the resources or infrastructure to adopt and implement DT technologies. **Regulatory hurdles:** Challenges in regulatory approval and validation of digital health twins for clinical use.
[25]	**Precision dosing:** DTs can be used to personalize chemotherapy and radiotherapy dosing for cancer patients, reducing toxicity and improving effectiveness. **Integration of multi-omics data:** DTs can integrate genomic, transcriptomic, and proteomic data for better precision in treatment planning.	**Data sparsity:** Lack of comprehensive and high-resolution patient data limits the ability of DTs to accurately model cancer progression and therapy responses. **High computational resources:** Generating and updating DTs requires significant computational power, which may not be feasible in all clinical settings.
[26]	**Multi-modal data integration**: The ability of DTs to combine imaging, genomics, and clinical data can provide a more holistic view of cancer progression, enhancing personalized treatment strategies. **Improved accuracy:** Mathematical modeling can improve the precision of DTs in predicting tumor behavior and therapeutic responses.	**Data integration issues:** Integrating multi-modal data (e.g., imaging, genomics) is complex and may result in inaccurate or incomplete models. **Biological complexity**: Capturing the heterogeneity of cancer through DTs is difficult, especially when accounting for tumor mutations and microenvironment variations.
[27]	**Agent-based modeling**: The use of computational frameworks based on DTs can simulate patient-specific treatment plans using agent-based models, which predict tumor growth and response to therapies. **Precision in treatment strategies:** DTs improve the ability to personalize treatment plans and optimize therapy efficacy for individual patients.	**Computational resource requirements**: DTs demand high computational power for simulations, especially for large-scale clinical use. **Data dependency:** The accuracy of DTs heavily depends on high-quality patient-specific data, which may not always be available.
[28]	**Spatially resolved DTs**: The development of generative DTs that can simulate tumor behavior in a 3D spatial context offers more accurate predictions of tumor growth and treatment responses. **Data-driven insights**: DTs can provide deeper insights into tumor biology, helping oncologists tailor treatment strategies effectively.	**Challenges in data integration**: Combining clinical, imaging, and genomic data to create spatially accurate DTs is a complex task. **Tumor heterogeneity**: Cancer’s inherent biological variability complicates the development of universal DTs for different tumor types.
[29]	**Radiotherapy personalization**: DTs allow for the simulation of radiotherapy treatment plans that are tailored to the individual patient’s tumor and surrounding tissues, minimizing normal tissue damage. **Tumor control optimization**: DTs can predict the efficacy of different radiotherapy protocols, enhancing treatment outcomes.	**Patient response variability**: Variations in how different patients respond to radiotherapy can challenge the effectiveness of DT-based simulations. **Dependence on advanced imaging**: Accurate tumor modeling through DTs requires high-quality imaging techniques, which may not be universally available.
[30]	**Immunotherapy optimization**: DTs can help personalize immuno-oncology treatments by simulating the immune response and optimizing therapeutic protocols based on patient-specific data. **Targeted immunotherapy approaches**: DTs can simulate tumor–immune interactions, enabling more targeted immunotherapies for specific patient profiles.	**Limited immune data**: Accurate DT models require detailed immune system data, which is often sparse or difficult to standardize. **Difficulty in immune simulation**: Simulating immune responses is inherently complex, and current models may not fully capture immune system dynamics.
[31]	**Theranostic digital twins (TDTs)**: These DTs can be used to personalize radiopharmaceutical therapies, minimizing toxicity while optimizing treatment efficacy based on patient-specific data. **Personalized therapeutic optimization**: TDTs can adjust treatment strategies for radiopharmaceutical therapies, enhancing precision and minimizing side effects.	**Data quality and quantity**: Reliable data on radiopharmaceutical interactions with tumors is needed to optimize the efficacy of TDTs. **Clinical implementation challenges**: Scaling the use of TDTs in clinical practice requires overcoming significant barriers in regulatory approval and clinical validation.
[32]	**Mechanistic learning**: Integrating mechanistic learning with DTs could improve decision-making processes by providing insights into cancer progression and response to treatment. **Improved clinical outcomes**: DTs hold the potential to bridge the gap between preclinical research and real-world clinical outcomes, leading to better therapeutic results.	**Complex data integration**: DTs require the integration of multiple data types (e.g., clinical, genomic, and imaging), which can be challenging and may lead to inconsistencies. **Clinical validation difficulty**: Ensuring that DT models align with actual patient outcomes in diverse oncology settings is a significant hurdle.
[33]	**Transformative potential**: DTs can revolutionize personalized oncology treatments by integrating patient-specific data with computational models for more accurate therapy predictions. **Long-term use in precision oncology**: The long-term goal of DTs in oncology is to develop more precise, efficient, and individualized treatment plans for cancer patients.	**Privacy concerns**: The large-scale use of patient data in DT models raises privacy and ethical issues, particularly with sensitive health information. **Data integration hurdles**: Integrating diverse datasets (genomic, clinical, imaging) while maintaining data privacy is a key challenge for DTs.
[34]	**Personalized clinical trials:** DTs can help design clinical trials that are more personalized, integrating real-time data assimilation and mathematical models to enhance patient recruitment and therapy customization. **Optimizing clinical trial outcomes**: By using DTs to simulate patient responses, clinical trial success rates may be improved.	**Real-time data integration**: Incorporating real-time data into DT models presents significant computational challenges and can affect model accuracy. **Resource-intensive simulations**: The computational costs of running personalized simulations for clinical trials may limit widespread adoption.
[35]	**Model-informed precision medicine**: Integrating AI/ML with DTs enhances the precision of oncology treatments, leading to better-targeted drug development and more effective patient care. **Clinical trial design optimization**: AI-powered DTs can help design more efficient and personalized clinical trials.	**Data integration complexity:** Using diverse, real-world data for AI/ML applications in DTs can complicate model accuracy and reliability. **Scalability and infrastructure needs**: High computational power is required for deploying AI/ML-driven DTs across clinical systems.
[36]	**Personalized oncology treatments**: Medical imaging and tissue-scale mathematical modeling are used to develop patient-specific DTs, improving tumor treatment personalization. **Simulation of tumor dynamics**: DTs can simulate tumor growth and response to therapy, enhancing treatment precision.	**Advanced imaging requirements**: DTs require high-quality imaging data to ensure accurate tumor modeling and predictions. **Complexity of modeling tumor dynamics:** Capturing the diverse and dynamic nature of tumors in a digital model is a significant challenge.
[37]	**Hybrid AI/Mechanistic models**: Combining AI with mechanistic modeling for DTs can improve therapy precision in oncology and other medical areas, like inflammatory bowel disease (IBD). **Improved therapeutic response:** Hybrid models can provide more accurate predictions of patient outcomes, leading to better-targeted therapies.	**Data integration challenges:** Integrating AI and mechanistic models requires diverse datasets and poses significant challenges in terms of consistency and accuracy. **Computational burden:** Hybrid models require substantial computational resources to simulate and predict patient outcomes accurately.
